# Putative Nanobacteria Represent Physiological Remnants and Culture By-Products of Normal Calcium Homeostasis

**DOI:** 10.1371/journal.pone.0004417

**Published:** 2009-02-09

**Authors:** John D. Young, Jan Martel, Lena Young, Cheng-Yeu Wu, Andrew Young, David Young

**Affiliations:** 1 Laboratory of Nanomaterials, Chang Gung University, Gueishan, Taiwan, Republic of China; 2 Department of Biochemistry and Molecular Biology, Graduate Institute of Biomedical Sciences, Chang Gung University, Gueishan, Taiwan, Republic of China; 3 Research Center of Bacterial Pathogenesis, Chang Gung University, Gueishan, Taiwan, Republic of China; 4 Adjunct Faculty, Laboratory of Cellular Physiology and Immunology, The Rockefeller University, New York, New York, United States of America; 5 Harvard University, Cambridge, Massachusetts, United States of America; University of California Merced, United States of America

## Abstract

Putative living entities called nanobacteria (NB) are unusual for their small sizes (50–500 nm), pleomorphic nature, and accumulation of hydroxyapatite (HAP), and have been implicated in numerous diseases involving extraskeletal calcification. By adding precipitating ions to cell culture medium containing serum, mineral nanoparticles are generated that are morphologically and chemically identical to the so-called NB. These nanoparticles are shown here to be formed of amorphous mineral complexes containing calcium as well as other ions like carbonate, which then rapidly acquire phosphate, forming HAP. The main constituent proteins of serum-derived NB are albumin, fetuin-A, and apolipoprotein A1, but their involvement appears circumstantial since so-called NB from different body fluids harbor other proteins. Accordingly, by passage through various culture media, the protein composition of these particles can be modulated. Immunoblotting experiments reveal that antibodies deemed specific for NB react in fact with either albumin, fetuin-A, or both, indicating that previous studies using these reagents may have detected these serum proteins from the same as well as different species, with human tissue nanoparticles presumably absorbing bovine serum antigens from the culture medium. Both fetal bovine serum and human serum, used earlier by other investigators as sources of NB, paradoxically inhibit the formation of these entities, and this inhibition is trypsin-sensitive, indicating a role for proteins in this inhibitory process. Fetuin-A, and to a lesser degree albumin, inhibit nanoparticle formation, an inhibition that is overcome with time, ending with formation of the so-called NB. Together, these data demonstrate that NB are most likely formed by calcium or apatite crystallization inhibitors that are somehow overwhelmed by excess calcium or calcium phosphate found in culture medium or in body fluids, thereby becoming seeds for calcification. The structures described earlier as NB may thus represent remnants and by-products of physiological mechanisms used for calcium homeostasis, a concept which explains the vast body of NB literature as well as explains the true origin of NB as lifeless protein-mineralo entities with questionable role in pathogenesis.

## Introduction

Nanobacteria (NB), bacterial entities with unusually small sizes and widespread distribution in animals and humans [1], [2], have been implicated in numerous diseases and as infectious agents associated with body fluids, blood infusion products, and vaccines [3]–[7]. These same entities have been linked to the earlier “nannobacteria” [8], [9] found in geological samples as well as fossil forms found on meteorites [10], indicating that they may represent primitive or overlooked life precursors. NB are controversial due to both their small size (50–500 nm) and marked pleomorphism, features that are currently not supported by conventional microbiology (see ref. 11 for an excellent critical review).

Other laboratories had earlier failed to culture NB as living entities [12]–[14], but some recent reports have confirmed an organismic origin for NB propagated from human pathological tissues based on the presence of DNA, synthesized proteins (some of which deemed to be bacterial in origin), and immunodetection of NB antigens [15]–[18]. While lacking direct evidence, a pathogenic role for NB has nonetheless been suggested for an alarming number of diseases based solely on morphological detection of NB, their staining by antibodies deemed specific for NB, and the demonstrated ability of such particles to propagate in cell-free media [refs. 3]–[7; see ref. 11 for a list of diseases that have been linked to NB].

Distinct features of NB include their small sub-micrometer sizes, slow growth, ability to change shapes under various culture conditions (pleomorphism), association with carbonate hydroxyapatite (HAP), formation of bio-membranes, and presence of a protein coating [1]–[6]. NB have been shown to grow in both the presence and absence of serum. In the presence of serum, NB tend to assume coccoid-like shapes, while in the absence of serum, NB proliferate more slowly but tend to be larger, producing so-called “igloos” or “shelters” with hollow interiors that appear to harbor coccoid-like NB inside them [1], [2]. Alternative mechanisms attempting to explain some of these NB features have been proposed [12], [19], [20]. In the study by Cisar et al. [12], NB were shown to originate from the nucleation of self-propagating HAP triggered by lipids like phosphoinositol. These authors further showed that NB, when cultured *in vitro* without serum, displayed a simple protein profile that differed markedly from the kind of complex coating normally associated with living microorganisms, implying that the NB-associated proteins might have been derived from apatite-binding proteins present in saliva.

Raoult et al. [19] have proposed instead that NB are “fetuin-mineralo complexes” that they call “nanons.” These authors showed that NB cultured in the absence of serum displayed no more than three protein bands, the main one being fetuin (here referred to as fetuin-A). Like Cisar et al. [12], they attributed this simple protein profile to the absence of non-specific, steric binding by serum proteins [19]. Both studies exploited the use of serum-free conditions to simplify the protein profile of cultured NB. The study by Raoult et al. [19] makes significant advances to our understanding of NB biology by conferring at last some biochemical specificity to the previously elusive NB entity and by linking NB for the first time to the biology of fetuin-A, a potent inhibitor of extraskeletal calcification and apatite formation [21], [22] that has recently been shown to form colloidal complexes with calcium and phosphate [23]–[27]. However, it is unclear whether fetuin-A is a strictly necessary component or “nucleator” of all NB reported to date, including NB derived in serum-free conditions or under conditions in which there is potentially little or no fetuin-A present.

Our own study [20] showed that simple amorphous calcium compounds like calcium carbonate produce self-propagating nanobacteria-like particles (NLP) that mimic many of the morphological features of NB. NLP formation is facilitated by calcium-binding proteins (e.g., albumin) and other divalent cations (Mg^2+^) [20]. Our study suggested that an amorphous calcium compound could in principle serve as a scaffold needed for further calcium-carbonate-phosphate crystallization, ultimately yielding carbonate HAP, considered the hallmark of NB. We also showed that monoclonal antibodies, marketed as NB-specific and used widely as markers for NB infection, react strongly with human serum albumin (HSA), calling in question their specificity as well as the nature of the protein coating associated with NB [20]. Both serum fetuin-A [19] and albumin [20] may thus be associated with NB, with their relative participation being dependent on the source of serum material used for obtaining NB (e.g., HS in our study vs. FBS, or fetal bovine serum, in Raoult et al's). In line with earlier studies that have also shown NB-like morphologies associated with geological samples enriched for simple calcite, carbonate, and silicate phases [28]–[31], our report cautions against the use of morphological and immunological criteria as the sole basis for assigning life to structures bearing resemblance to microorganisms and other biological entities.

These studies illustrate the complexities of the NB phenomena seen with the different sources of material used as well as the lack of biochemical parameters that can be used to define and characterize NB insofar as their formation and ambiguous chemical composition and growth characteristics are concerned. In fact, it is not clear whether NB represent single or multiple biological entities, but these studies [12], [19], [20] as well as earlier reports [32], [33] suggest that NB may represent mineralized proteinaceous complexes rather than living organisms. That is, the NB HAP may in fact be associated with calcified proteins [33]. In line with these observations, the original discoverers of NB have later revised the term NB to “nanoforms” [32] and have more recently suggested the use of “calcifying nanoparticles” (or CNP) instead of NB [3], [4], [7]. However, in spite of their questionable status as live organisms, CNP continue to be viewed as transmissible and pathogenic agents of renal and arterial calcifications as well as an expanding number of other diseases [3], [4], [7], [34]–[37].

Instead of considering NB as the cause of pathological or infectious anomalies, we propose that NB may be common physiological remnants linked to normal calcium homeostasis. We demonstrate here that the same amorphous mineral complexes composed of simple calcium compounds and well-known proteins, that normally prevent calcium from crystallizing, can also behave as scaffolds for crystallization once the same inhibition is overcome. Accordingly, NB may be molded or morphed by multiple calcium-binding proteins and ions present in the milieu, allowing them to mirror the environment and to vary in composition according to the environmental imprint. We believe that this alternative concept allows for explaining the entire published phenomenology of NB, including their complex protein composition, HAP coating, and pleomorphism. Viewed this way, NB are no more than by-products of the normal, homeostatic mechanisms found in the body and throughout nature designed to deal with excess calcium, phosphate and other ions, and these same mechanisms serve to modulate the final composition of NB.

## Results and Discussion

### General strategy and working hypothesis

The biochemical characterization of NB is hampered by their slow propagation in culture, with doubling times of 3 days or more [1]–[6] and a complete lack of understanding of what determines exactly the NB composition. Thus, rather than just using published protocols to study NB as complex and ambiguous entities derived in culture, we also attempted a reverse strategy by first generating well-defined amorphous mineral complexes—particles that we have labeled NLP to distinguish them from the published NB. That is, we sought to reconstitute NLP using simple precipitation methods by adding calcium, carbonate, magnesium, and phosphate to various well-defined cell culture media, followed by our demonstration that these NLP are virtually identical to the NB obtained using published protocols [1]–[6]. This approach can be justified by the fact that all NB cultures to date have originated from the introduction of some exogenous factor(s), be them serum, some other kind of body fluid, or pulverized tissue homogenates, to normal culture media that may very well have perturbed the local equilibrium of calcium, resulting in slow and ambiguous calcium and HAP precipitations that are then described as NB [1]–[6]. By our adding, in a controlled manner, the various ions to exceed their saturation levels, the process of NB formation can be enhanced many fold, resulting in more quantifiable and reproducible observations. This alternative approach allows for the systematic testing and monitoring of a large number of variables relevant to NB formation which otherwise would have been difficult to study. Once the main components of NB were defined through this *in vitro* reconstitution approach, we were then able to simulate, and even accurately predict, the composition of NB as a result of environmental changes as well as to reconcile our findings with the entire body of published data on NB.

Central to our hypothesis is the currently accepted notion that extraskeletal bio-mineralization or calcification is thermodynamically favored at concentrations of calcium and phosphate normally found in body fluids [21]–[27], [38]. That is, with regards to bio-mineralization, given this well-known propensity for calcification under normal conditions, the key challenge appears not so much understanding how calcifications are triggered, but how they are inhibited! In this context, competing ions, like carbonate and magnesium, and calcium-binding proteins, like fetuin-A and albumin, should probably be viewed as calcification inhibitors. However, these same calcification inhibitors, following their initial binding to calcium and/or phosphate, if not disposed, can be overcome eventually by an excess of calcifying ions found in certain body fluids or culture conditions and, as such, they may become constituents of the calcified end-product. According to this alternative concept, the same inhibitors of calcium or apatite crystallization which are deployed physiologically to inhibit calcification may provide a nucleating seed or nidus for NB formation once it is overcome by the presence of calcium and/or phosphate found normally in high concentrations in culture or body fluids. In the final analysis, it is this same calcium or apatite inhibitory complex that provides the semblance of a filterable and transferable infectious agent characterized as NB.

### Inhibitory effects of serum on NB formation

**Figure 1:**
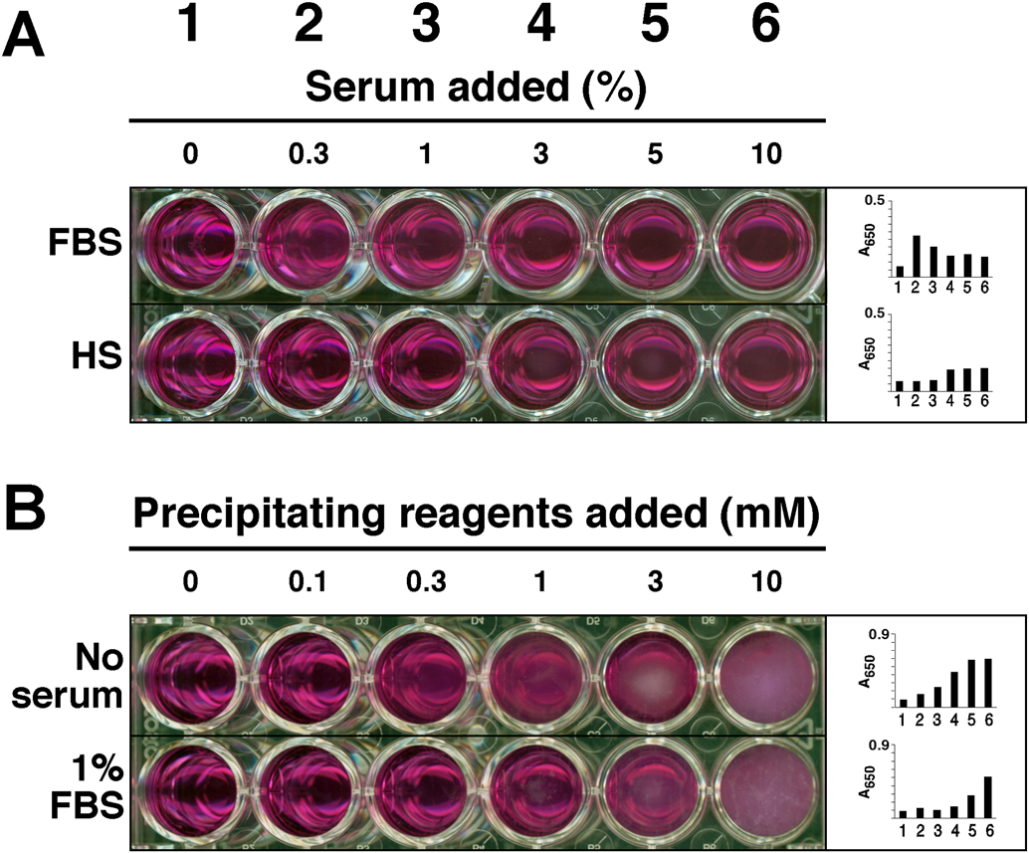
Culture of NB from serum and formation of NLP. (A) Culture of NB from FBS (top panel) and HS (bottom panel). Serum was inoculated into DMEM to the indicated concentrations. After incubating for 1 month at 37°C, NB were detected by visual inspection and A_650_ (insets). For FBS, the amount of NB was maximal at 0.3%, decreasing thereafter with higher FBS concentrations, while for HS, NB were only visually noticeable at HS concentrations exceeding 3%. (B) Formation of NLP in DMEM. NLP were prepared by successively adding solutions of 0.25 M CaCl_2_, Na_2_CO_3_, and NaH_2_PO_4_ (pH 7.4) into DMEM as indicated and incubated at 37°C overnight, which resulted in dose-dependent formation of NLP (top row). The same experiment was repeated in the presence of 1% FBS, which showed inhibition of NLP formation (bottom row). This serum inhibition was overcome by increased concentrations of precipitating reagents, at 3 mM and above.

To date, the most widely used published procedure for NB formation consists simply of inoculating a source of NB, presumably serum, another body fluid, or a tissue homogenate, into culture medium (usually Dulbecco's Modified Eagle's Medium or DMEM) supplemented with or without added serum [1], [2]. Here, we adapted this procedure to 24 well plates, followed by incubation at 37°C under controlled cell culture conditions and periodic visual observation, phase microscopy, or A_650_ reading using a spectrophotometer. Figure 1A illustrates one such experiment, done with varying amounts of FBS or HS, along with a demonstration of the intrinsic problems that this kind of procedure harbors. The major problem with all such cultures is the long incubation time needed for NB formation, which in the case of medium inoculated with serum, may take over several weeks for any detectable precipitation. In fact, the NB data generated for Figure 1A, showing barely detectable precipitation of particles in the presence of various amounts of serum, required one month of incubation at 37°C, producing high margin of error and results that are ambiguous, at best, even when carefully collected. Furthermore, while giving all the morphological features published earlier for NB [1]–[6], the material generated after this long period of incubation would have been hardly sufficient for any biochemical characterization.

Nonetheless, the serum dose-dependence experiment displayed in Figure 1A, while giving marginal amounts of precipitation even after one month of incubation, demonstrates an intriguing pattern. We noticed that amounts of FBS exceeding 0.3% were generally inhibitory on NB formation (Fig. 1A, “FBS” row) whereas HS was only mildly inhibitory at much higher levels, usually at 3% or more (Fig. 1A, “HS” row). That is, in the case of HS, the amount of precipitates increased slightly with an increase in the amount of serum inoculated into the medium, reaching a plateau or decreasing thereafter with further increases in serum concentration. The exact amount of inhibition appeared to vary with the lot or batch of FBS and HS used, with significant scatter and margin of error, but the dual effects of enhancement of NB growth at low amounts of serum followed by inhibition at higher serum levels appeared to be generally reproducible with all the serum samples tested to date.

These results were surprising since serum was the first source of NB described in the literature [1]–[6]. As demonstrated by these experiments, the difficulty with working with serum alone, resulting in marginally detectable levels of particle precipitation even over a prolonged period of time, reflects the inherent complexities and ambiguities involved. In retrospect, these same ambiguities can easily lead the observer to overlook important observations, as was the case here with serum inhibition, a paradoxical result given the fact that serum had earlier been described as the source of NB that led to their discovery.

### Formation of NLP using simple precipitation procedures

In order to have a better controlled, biochemical handle on NB-like formations, we next turned to alternative methods for generating NB-like precipitations, which we have called NLP. Using precipitation methods outlined in the *Materials and Methods*, various calcium-containing NLP were obtained in DMEM, in the presence or absence of serum, through the simple addition of calcium, magnesium, carbonate and/or phosphate to concentrations at or exceeding saturation levels (0.3 to 10 mM for calcium, carbonate, and phosphate). The amount of precipitation could be controlled through the amount of precipitating reagents inoculated into DMEM, as seen by visual inspection and turbidity measurement at 650 nm (Fig. 1B). It should be noted that DMEM alone has 1.8 mM calcium, 0.9 mM phosphate, 0.8 mM magnesium, and 44 mM carbonate. The fact that adding only 0.3 mM calcium, carbonate, and phosphate to DMEM could induce precipitation visible to the eyes (Fig. 1B, “No Serum” row, well 3) suggests that the DMEM composition must be already close to saturation levels of calcium and phosphate. NLP could also be formed in the presence of serum, illustrated by the experiment shown in Fig. 1B (2^nd^ row) that used 1% FBS while varying the amount of precipitating ion reagent added. As demonstrated earlier with NB, the addition of this 1% FBS was clearly inhibitory on NLP formation, requiring as much as 3 mM each of calcium, carbonate, and phosphate to overcome the inhibition (compare visual reading and A_650_ data obtained between the various wells of the two rows).

**Figure 2:**
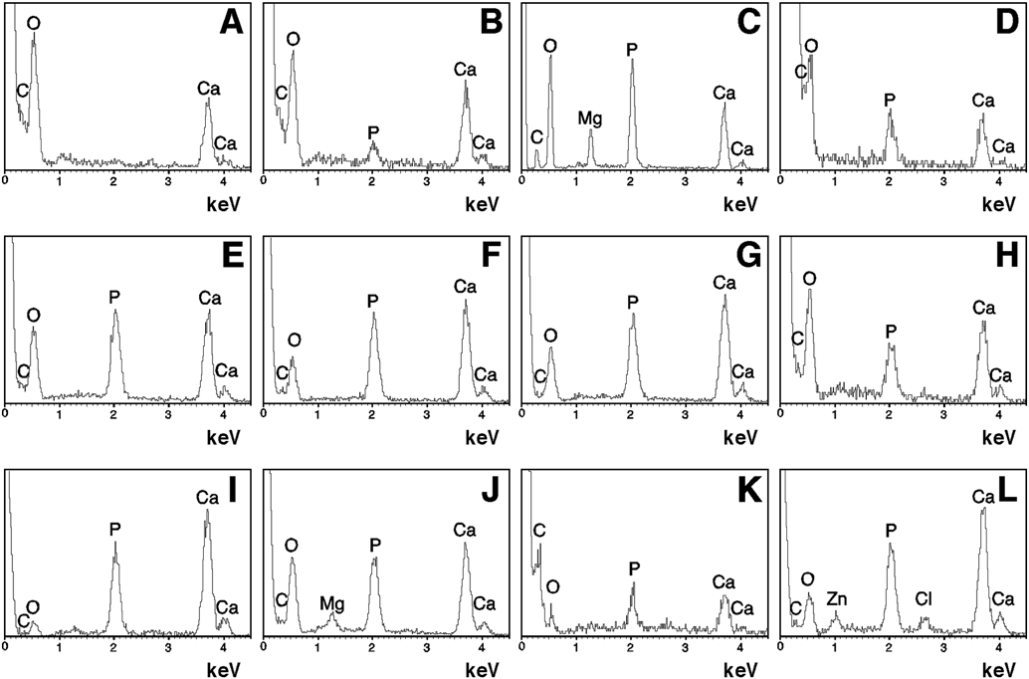
Energy-dispersive X-ray spectroscopy of NLP and NB. EDX results were selected to illustrate the wide spectrum of NLP compositions, with respect to the amounts of calcium and phosphorus incorporated, and their similarities to NB. (A) Elemental composition of NLP prepared by adding CaCl_2_ and (NH_4_)_2_CO_3_ at 50 mM into DMEM corresponding to calcium carbonate. (B and C) Amorphous CaCO_3_ NLP produced as in (A) except that the precipitating reagents were added to DMEM and incubated at room temperature for 2 days (B) or one month (C), showing increased incorporation of P with prolonged incubation. Ca/P ratios are 5.09 for (B) and 1.60 for (C), with the latter close to the theoretical value of 1.67 associated with stoichiometric HAP. (D–H) Formation of NLP with various inputs of ions, including P, yielding different Ca/P ratios. (D) NLP formed from 0.25 M each of CaCl_2_, Na_2_CO_3_, and NaH_2_PO_4_, mixed in with DMEM at vol. ratios of 2∶5∶5∶13 that had been incubated in DMEM for 2 days, revealing the presence of magnesium ions and a Ca/P ratio of 0.78. (E–H) NLP were prepared like in (D) at vol. ratios of (E) 1∶1∶1∶22, yielding a Ca/P ratio of 1.29; (F) 3∶2∶2∶43, Ca/P ratio of 1.49; (G) 9∶5∶5∶106, Ca/P ratio of 1.76; (H) 2∶1∶1∶21, Ca/P ratio of 2.03. NLP were submitted to EDX immediately after formation. (I–L) Elemental composition of various NB preparations. (I and J) NB obtained from one-month-old DMEM cultures containing (I) 10% HS or (J) 10% FBS. Ca/P ratios: 1.75 (I) and 1.53 (J). (K) NB strain DSM 5819, after one week in DMEM without serum, giving a Ca/P ratio of 1.45. (L) “Nanons” after one week in DMEM; Ca/P ratio: 1.66.

The NLP precipitates could be sedimented by centrifugation, washed, and transferred to new medium, or allowed to grow in size for various incubation times prior to their transfer. These NLP could be made to vary in ionic composition by simply changing the amounts of calcium, carbonate, and phosphate, as well as serum, added to the precipitation mixture, as demonstrated by energy-dispersive X-ray spectroscopy (EDX; Fig. 2). In our hands, a wide repertoire of particle compositions could be obtained, ranging from predominantly calcium carbonate or calcium phosphate particles to calcium particles containing carbonate, magnesium, and phosphate in various proportions (Fig. 2A–H).

Using this precipitation procedure, the effects of the various reagents (e.g., ions, serum, trypsin, and EDTA) on the formation of NLP could be studied either independently or in concert. By visual inspection, it was obvious that calcium-containing NLP enriched either in carbonate or phosphate differed markedly from each other, with the carbonate-enriched NLP appearing more chalky and granular, and being easier to disperse in aqueous solutions, while the phosphate-enriched NLP appeared gray and gelatinous, and were much harder to disperse. When incubated in medium, the phosphate-enriched NLP grew much faster than the carbonate-NLP, with the latter showing a finer and more granular precipitate than the phosphate-enriched NLP. Furthermore, NLP that had been formed in relatively high amounts of serum (5–10%) displayed a finer and more granular structure when compared with NLP formed in the complete absence or low amounts of serum (less than 1%), which appeared as coarse aggregates.

### Chemical composition of NLP and NB and acquisition of phosphate and HAP by amorphous calcium complexes

Irrespective of their initial composition, all calcium-based NLP samples (102 tested to date) could be shown by EDX to acquire phosphorus following incubation in DMEM (Fig. 2B–H). The rates of P acquisition were both time and temperature dependent, and at 37°C, acquisition reached stable calcium:phosphorus ratios (or Ca∶P ratios) between 1 and 2 Ca to 1 P, after a 1–3 day incubation, with the final Ca∶P ratio eventually becoming independent of the initial Ca∶P ratios used to form NLP. That is, even amorphous calcium carbonate particles that had been formed in the absence of phosphate (Fig. 2A) could be shown to avidly bind P and to gradually convert to calcium phosphate (Fig. 2B and C). On the other hand, NLP that had been formed in the presence of phosphate also showed marked variability in the final P concentrations when examined by EDX (Fig. 2D–H). This variability seen with the Ca∶P ratios, which differs significantly from the stoichiometric Ca∶P ratio of 1.67 associated with pure HAP, appears to be a common finding linked to the structure of bone and have been attributed to amorphous calcium carbonate and amorphous phosphate intermediates as well as small and incompletely crystallized carbonate-HAP [38], [39]. Within the same batch of experiments, we noticed marked variability and a great margin of error for Ca∶P ratios, especially when the same samples were exposed to different temperatures (room temperature, 4°C, or 37°C). P absorption was detected immediately after the 30 minutes of incubation used to form NLP and appeared to increase steadily with time, followed generally by sharp increases within short spans of time that typically occurred between 1 hour and several days depending on the incubation temperature. This variability seemed to contribute to the large margin of error seen.

These findings showing incorporation of P by NLP could be identically reproduced with both human and bovine NB (Fig. 2I and J) that had been obtained using published protocols [1], [2], as outlined in the *Materials and Methods*, and with characterized NB strains (Fig. 2K and L). The latter include three strains of *Nanobacterium sanguineum* that were deposited by Kajander with the German Collection of Microorganisms (DSM 5819–5821) and the *Nanobacterium* sp. strain Seralab 901045 used by Raoult et al. [19] and that they have called “nanons.” As with NLP, Ca∶P ratios for NB ranged between 1-to-2 Ca to 1 P and showed marked variability within the same strain or type of NB depending on the number of days of incubation, the initial inoculum size, and whether serum was present or not (not shown). Like their NLP counterpart, NB that had been subcultured in 10% FBS also showed finer and more granular precipitates as compared with those cultured without serum or with low amounts of this supplement.

**Figure 3:**
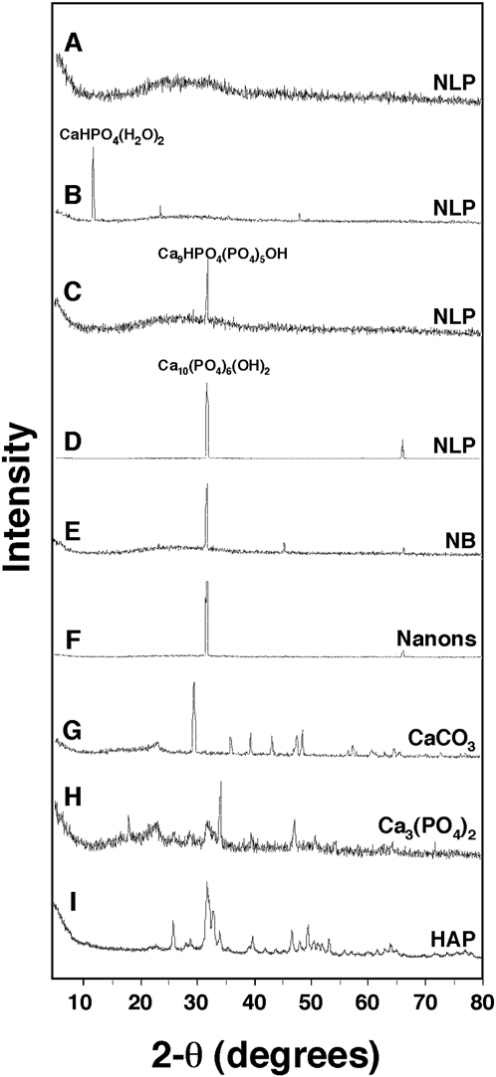
Powder X-ray diffraction analysis of NLP and NB. (A–D) Various amorphous and crystalline patterns are associated with NLP. NLP were prepared from the inoculation of 3 mM each of CaCl_2_, Na_2_CO_3_, and NaH_2_PO_4_ into DMEM. NLP were examined by XRD (A,B) immediately after formation, which revealed either an amorphous pattern (A) or the presence of the mono-calcium form CaHPO_4_(H_2_O)_2_, or they were examined after the following periods of incubation in DMEM at 37°C: (C) 1 day; and (D) 4 days. (E) NB obtained from DMEM containing 10% HS. (F) “Nanons” after one week in DMEM without serum. Peaks were assigned the specified chemical structure after comparing peaks with the database. With the exception of (C), which revealed the Ca_9_ form of HAP, the peaks seen in (D–F) all corresponded to Ca_10_ HAP. Peaks at 32.0 degrees and 31.8 degrees correspond to the Ca_9_ and Ca_10_ forms of HAP, respectively. Notice also the small peaks at 66.2 degrees seen only with the Ca_10_ form (D–F). Commercial powders of (G) CaCO_3_, (H) Ca_3_(PO_4_)_2_, and (I) HAP were included as controls.

Conversion to HAP could be ascertained by X-ray diffraction (XRD) analysis (Fig. 3), which also revealed a time and temperature dependent process. Immediately after formation, some of the NLP complexes revealed either amorphous patterns with no discernible crystalline peaks (Fig. 3A) or mono-calcium phosphate peaks (Fig. 3B). Following incubation at room temperature or 37°C, peaks eventually shifted to correspond to larger crystalline forms, reaching eventually Ca_9_ calcium phosphate compounds or Ca_10_ compounds corresponding to HAP within 1–2 days (Fig. 3C and D; compare signals with the CaCO_3_, calcium phosphate, HAP controls shown in Fig. 3G–I). Accumulation of HAP showed marked variability as a function of time, with some samples displaying HAP peaks after only 30 minutes of incubation, but typically more extensive incubations varying between several hours to 2 days were needed for this conversion. Even at room temperature or at 4°C, conversion to HAP could still be seen, albeit with much slower kinetics (not shown). In fact, samples that had initially shown only amorphous patterns and that had been washed and suspended in water alone at 4°C also showed conversion to HAP when examined two weeks later (not shown). Based on serial observations made during regular intervals, there appeared again to be a rapid conversion to HAP within a short span of time; however, the onset of this sharp HAP conversion could not be defined accurately given the wide margin of error seen with each experiment.

Similar results were also obtained with both human and bovine NB cultured directly from serum-containing medium, with Figure 3E showing one representative XRD spectrum obtained for NB cultured from HS, revealing the presence of HAP. Several NB strains also showed HAP patterns that were virtually indistinguishable from those seen with NLP, as illustrated by Figure 3F, which shows one representative spectrum associated with “nanons,” the *Nanobacterium* sp. strain Seralab 901045.

**Figure 4:**
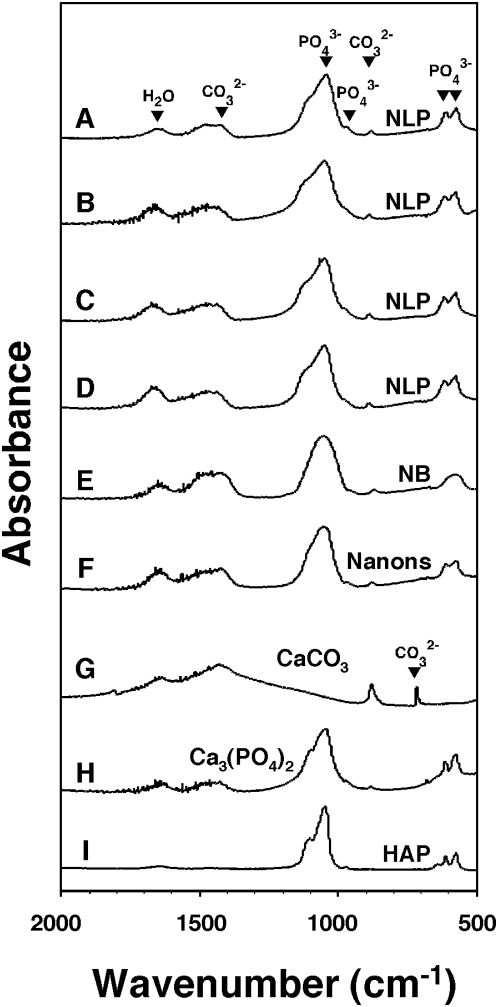
Fourier-transformed infrared spectroscopy of NLP and NB reveals the presence of both carbonate and phosphate groups. (A–D) NLP were prepared as in Fig. 3 and submitted to FTIR spectroscopy (A) immediately after formation, or after the following periods of incubation at 37°C in DMEM: (B) overnight; (C) one week; and (D) one month. The FTIR spectrum of NLP revealed peaks characteristic of carbonate ions at 875 cm^−1^ (ν2) and 1,430 cm^−1^ (ν3). Phosphate peaks were also present at 566 cm^−1^, 604 cm^−1^ (ν4), and between 1,033–1,100 cm^−1^ (ν3). The peak seen at 1,651 cm^−1^ was due to the presence of water in the sample. (E and F) NB showing FTIR spectra similar to those of NLP. (E) NB cultured from DMEM containing 10% healthy HS, after one month. (F) “Nanons” after one week in DMEM without serum. Commercial powders of (G) CaCO_3_, (H) Ca_3_(PO_4_)_2_, and (I) HAP were included as controls. CaCO_3_ produced an additional peak near 650 cm^−1^ attributed to calcite that was not found in our experimental samples.

The HAP of NB had been reported earlier as carbonate-HAP, similar to the bone mineral [2], [4]–[6]. It should be noted that HAP can not be distinguished from carbonate-HAP based on XRD alone since both spectra show overlapping peaks at 33 degrees on the 2-θ scale. On the other hand, Fourier-transformed infrared spectroscopy (FTIR) done on similar NLP specimens revealed peaks characteristic of the vibration modes of amorphous carbonate such as bending seen at 875 cm^−1^ as well as a split in the asymmetric stretching band seen at 1430 cm^−1^ (Fig. 4A–D; compare with signals obtained for CaCO_3_ as shown in Fig. 4G; see also references 40 and 41). Phosphate absorption bands corresponding to HAP were also seen at 564, 603, 955, and 1085 cm^−1^ in NLP specimens (Fig. 4A–D; compare with signals generated for calcium phosphate and HAP shown in Fig. 4H and I, respectively). Again, these FTIR spectra obtained for NLP were virtually identical to those obtained for NB cultured from HS (Fig. 4E) or for several NB strains (“nanons” shown in Fig. 4F).

**Figure 5:**
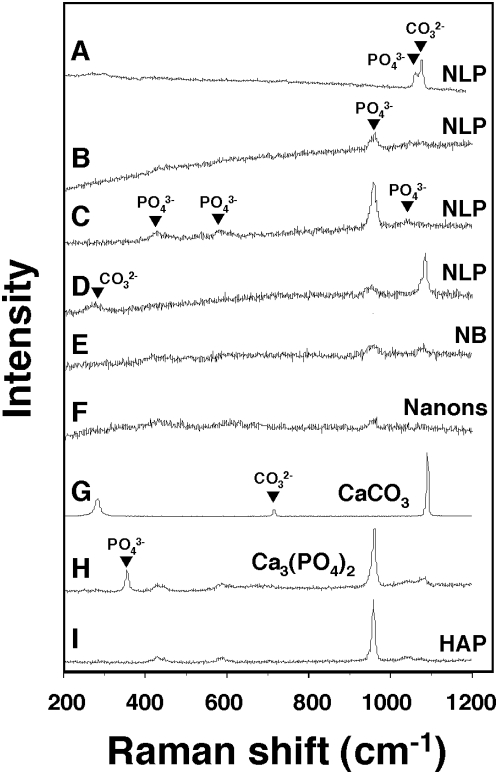
Micro-Raman spectroscopy of NLP and NB reveals the presence of both carbonate and phosphate. (A–D) NLP were prepared as in Fig. 3 and submitted to micron-Raman spectroscopy (A) immediately after formation, or after the following periods of incubation at 37°C in DMEM: (B) overnight and (C) one week. (D) NLP were prepared by addition of 10 mM each of Na_2_CO_3_ and (NH_4_)_2_CO_3_ in DMEM. Carbonate peaks were seen at 290 cm^−1^ and 1,070 cm^−1^, while phosphate peaks were seen at 440 cm^−1^, 581 cm^−1^, 962 cm^−1^, 1,048 cm^−1^, and 1,076 cm^−1^. The four NLP specimens show marked variability of peaks. (E) NB obtained from a culture of DMEM containing 10% HS reveal small peaks of phosphate at 440 cm^−1^ and 962 cm^−1^ as well as a carbonate peak at 1070 cm^−1^. (F) “Nanons” showing low peaks of phosphate at 440 cm^−1^, 581 cm^−1^, and 962 cm^−1^. Commercial powders of (G) CaCO_3_, (H) Ca_3_(PO_4_)_2_, and (I) HAP were included as controls.

The presence of amorphous calcium carbonate in NLP could be further demonstrated by Raman spectroscopy, which revealed a low broad peak at about 1080 cm^−1^ (more noticeable in Fig. 5A and D; see also refs. 41 and 42). Control calcium carbonate calcite crystals also revealed small peaks at 280 cm^−1^ and 712 cm^−1^ corresponding to crystalline calcite (Fig. 5G), but these peaks were rarely seen in our NLP samples (Fig. 5D). Some spectra also revealed phosphate ion peaks at 440 cm^−1^, 581 cm^−1^, 962 cm^−1^, 1,048 cm^−1^, and 1,076 cm^−1^ (Fig. 5A–D). The signal strengths attributed to carbonate or phosphate could be modulated by varying the input of carbonate versus phosphate used in the precipitation protocol, with Figure 5A–D illustrating a wide spectrum of possibilities (compare signals with the CaCO_3_, calcium phosphate, HAP controls shown in Fig. 5G–I). Like for NLP, the Raman spectra of NB also revealed marked variability, as illustrated by the spectra obtained for NB cultured from HS (Fig. 5E) and for “nanons” (Fig. 5F), which showed small peaks for carbonate and/or phosphate in different proportions depending on the culture conditions. The accumulation of phosphate, however, either in amorphous form or as HAP, appears to be favored over time, as illustrated by a gradual shift of the Raman spectra towards the phosphate signal at 962 cm^−1^.

**Figure 6:**
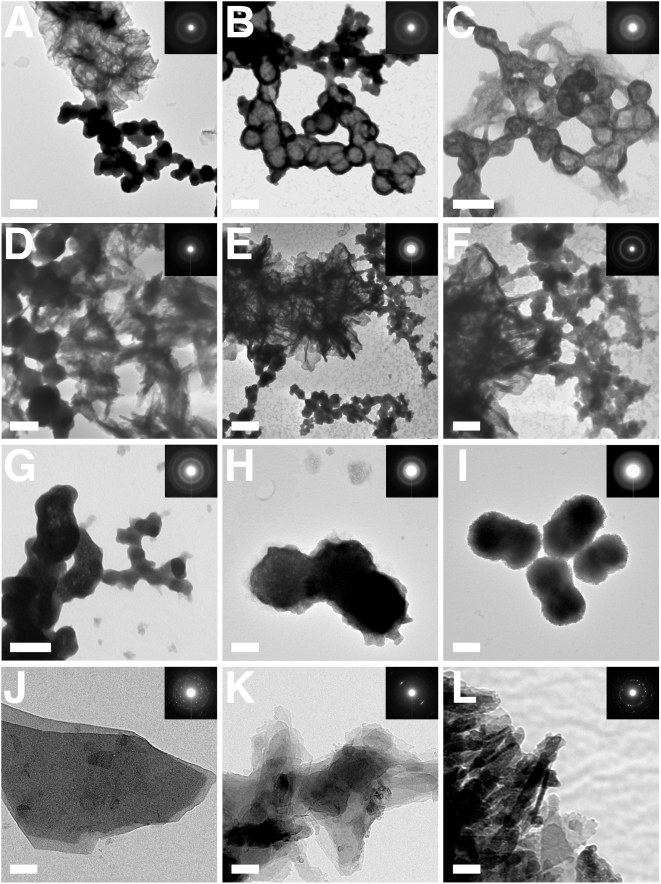
Negative-staining TEM and electron diffraction patterns of NLP and NB. (A–G) NLP were prepared as in Fig. 3 and submitted to TEM after incubation in DMEM at 37°C for either 2 days (A–C, G), 2 weeks (D), or 1 month (E and F). Round, dividing, or budding NLP are shown in close association with a crystalline phase, which shows either as a network of filamentous/membranous materials or as spindles/needles. The electron diffraction pattern characteristic of polycrystalline material obtained for both phases is shown in the inset. (B–D) show NLP with coccoid shapes and diameters smaller than 100 nm among larger particles. (D–F) Crystalline biofilms associated with NLP are seen with longer incubations. (F) is a magnified image of (E) depicting the transition between the round NLP and the crystalline matrix. (H and I) NB showing cell-dividing forms similar to NLP. (H) NB obtained from HS and (I) “nanons,” as in Fig. 3. Small crystalline projections can be distinguished on their surface. NB look virtually indistinguishable from NLP (compare with image G taken of NLP). Commercial (J) CaCO_3_, (K) Ca_3_(PO_4_)_2_, and (L) HAP produce a higher degree of crystallinity compared to NLP, NB, and “nanons” (insets). Scale bars: 100 nm (B–D, H–J, L); 200 nm (A, E–G, K).

The crystalline nature of both NLP and NB was characterized further by X-ray diffraction pattern obtained on selected specimen areas magnified by TEM (Fig. 6A–I and insets). NLP and NB produced similar morphologies that will be discussed in more detail in a later section (compare Fig. 6A–G obtained for NLP with Fig. 6H and I for NB). NLP, formed by precipitation using different inputs of carbonate and phosphate, produced X-ray diffraction patterns characteristic of polycrystalline material as shown by the presence of several concentric rings seen irrespective of the conditions used for their preparation (Fig. 6A–G). The patterns obtained for human NB (Fig. 6H) and the NB strain “nanons” maintained in FBS (Fig. 6I) also revealed similar fuzzy rings indicative of polycrystalline material. It should be noted however that NLP, NB, and “nanons” produced electron diffraction patterns with a lower degree of crystallinity compared to commercial lots of calcium carbonate, tri-calcium phosphate, and HAP (Fig. 6J–L). These commercial preparations revealed preferred angles of diffraction by means of characteristic arrays of dot (Fig. 6J–L) which could not be distinguished in the patterns obtained for NLP or NB (Fig. 6A–I). With continued incubation of NLP at 37°C for several days, there was a gradual shift toward crystalline patterns, an observation similar to that made with the other spectroscopies. That is, the patterns obtained for NLP were seen to gradually show an increase in the number and the intensity of the concentric rings. However, such patterns were still lacking in the arrays of dots typical of synthetic preparations of HAP (not shown), indicating the relatively low levels of crystallinity seen associated with NLP and NB. This low crystallinity seen for NLP, NB, and “nanons” is consistent with the presence of an amorphous phase of calcium carbonate and calcium phosphate in these nanoparticles. Similar patterns have been obtained for biological HAP found in the bone [43]. Such amorphous-crystalline interfaces had earlier been attributed to the incorporation of carbonate ions into HAP, a process thought to alter the molecular organization and structure of the resultant HAP complex [44].

Taken together, these results indicate that the amorphous calcium complexes constituting NLP invariably acquire phosphate and gradually convert to carbonate HAP, while retaining residual amounts of both amorphous carbonate and phosphate in their structure. Even amorphous calcium carbonate NLP left incubating in serum-free DMEM could be shown to convert to HAP, a result consistent with earlier findings demonstrating the transformation of CaCO_3_ [45]–[47] and CaCO_3_-containing nacre [48] into HAP when incubated in phosphate-containing solutions at room temperature. HAP formation is deemed to be autocatalytic [39] in that the developing HAP crystals propitiate the nucleation of additional HAP, siphoning the more soluble amorphous calcium complexes into the highly insoluble HAP product. In this sense, these same amorphous calcium complexes, once formed, appear to function as scaffolds for nucleation of HAP.

### Morphologies associated with NLP and NB and structural changes linked to phosphate and HAP incorporation

**Figure 7:**
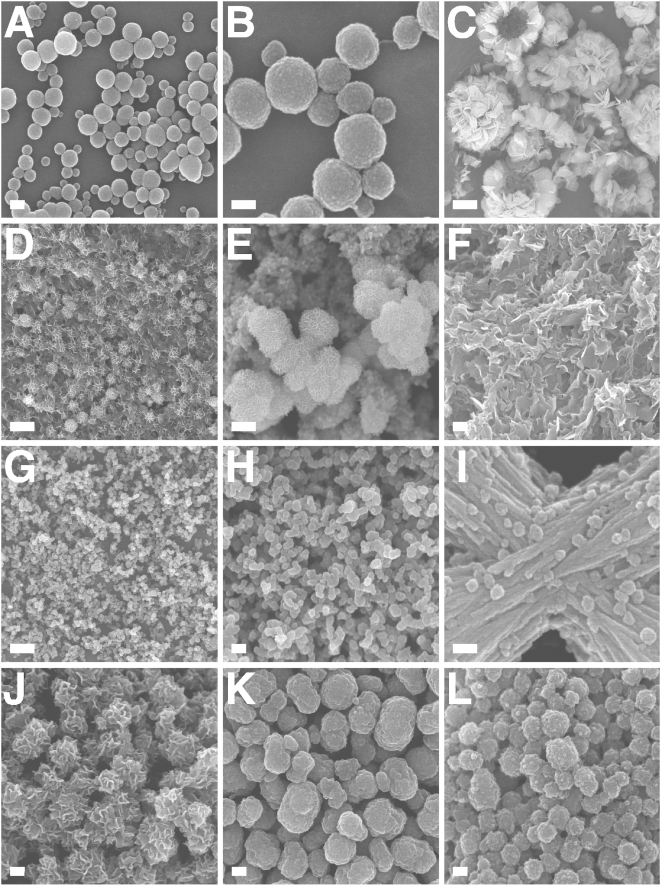
Identical morphologies of NLP and NB seen by field-emission SEM. (A–F) NLP were obtained as in Fig. 3 and incubated in DMEM for either 2 days (A and B), 4 days (D and E) or 1 month (C and F). (A) NLP consisted of spherical particles with a few dumbbell formations suggestive of cell division. (B) Enlarged image of NLP presented in (A) showing round particles with a size ranging 50–200 nm. (C) Conversion of round NLP to crystalline HAP in the form of needle-like formations along with large exposed hollow spheres in the shape of “igloos” and “shelter” forms of NB (see ref. 4). (D) Conversion of NLP into a crystalline film-like matrix. (E) Enlarged image of (D), showing crystalline surface of NLP, with their rough surface coated with small needle-like crystals. (F) Dense crystalline matrix formed after prolonged incubation of NLP. (G) Small-sized NLP obtained in the presence of excess magnesium and carbonate. NLP were prepared from the inoculation of 3 mM CaCl_2,_ 18 mM Na_2_CO_3_, 3 mM NaH_2_PO_4_, and 3 mM MgCl_2_ into DMEM. (H) Enlarged image of NLP seen in (G), showing sizes typically around 100 nm. (I) NLP prepared as in (A), showing round particles attached to filamentous and membranous material. (J–L) NB with morphologies similar to those of NLP. (J) NB cultured from HS, as in Fig. 6, consisting of coalesced particles covered with needle-like formations. (K) “Nanons” and (L) NB strain DSM 5819 cultured in DMEM for one week without serum, showing round, ellipsoid and division-like large formations. (L) NB strain DSM 5819. Scale bars: 100 nm (B, I); 200 nm (A, C, F, H, J–L); 1 µm (G); 2 µm (C); 5 µm (D).

To further ascertain the similarities between NLP and NB, a detailed morphological analysis was performed on the precipitates generated. The morphology of the various NLP obtained here was studied as a function of the various input components and compared to that seen with NB cultured from serum as well as several well-defined strains of NB. As evidenced by scanning electron microscopy (SEM) and transmission electron microscopy (TEM), incubation of amorphous calcium complexes in medium at 37°C produced a progressive change in shape from the round, coccoid-like particles with broad size distribution and formations reminiscent of cellular divisions (Fig. 6A–C and Fig. 7A and B) to a gelatinous, membrane-like precipitate similar to the biofilm described earlier in association with NB (Fig. 6D–F and Fig. 7D–F and I). We noticed that the NLP formed in the presence of phosphate were much larger (Fig. 7A and B), coalescing to form biofilms from the outset (Fig. 7D). Islands of HAP crystals in the shape of needles or spindles could also be seen (Fig. 6A, C, and D–F; Fig. 7C–F), resembling earlier descriptions of NB [1], [2]. In many of our NLP samples, amorphous particles could also be seen co-existing with film-like mattresses (Fig. 6A–F; Fig. 7D and I), with the round particles appearing to coalesce to form films. These same morphological features seen here with NLP, covering a wide spectrum of shapes, could be reproduced with NB obtained from serum (Figs. 6H and 7J) and NB strains (Figs. 6I and 7K and L). To date, all precipitates giving film morphologies have also revealed HAP peaks when examined by XRD, indicating that the film-like structures may represent stable crystalline forms derived from amorphous mineral particles. These results further support the notion that amorphous calcium compounds may serve as nuclei or nidi for HAP deposition.

**Figure 8:**
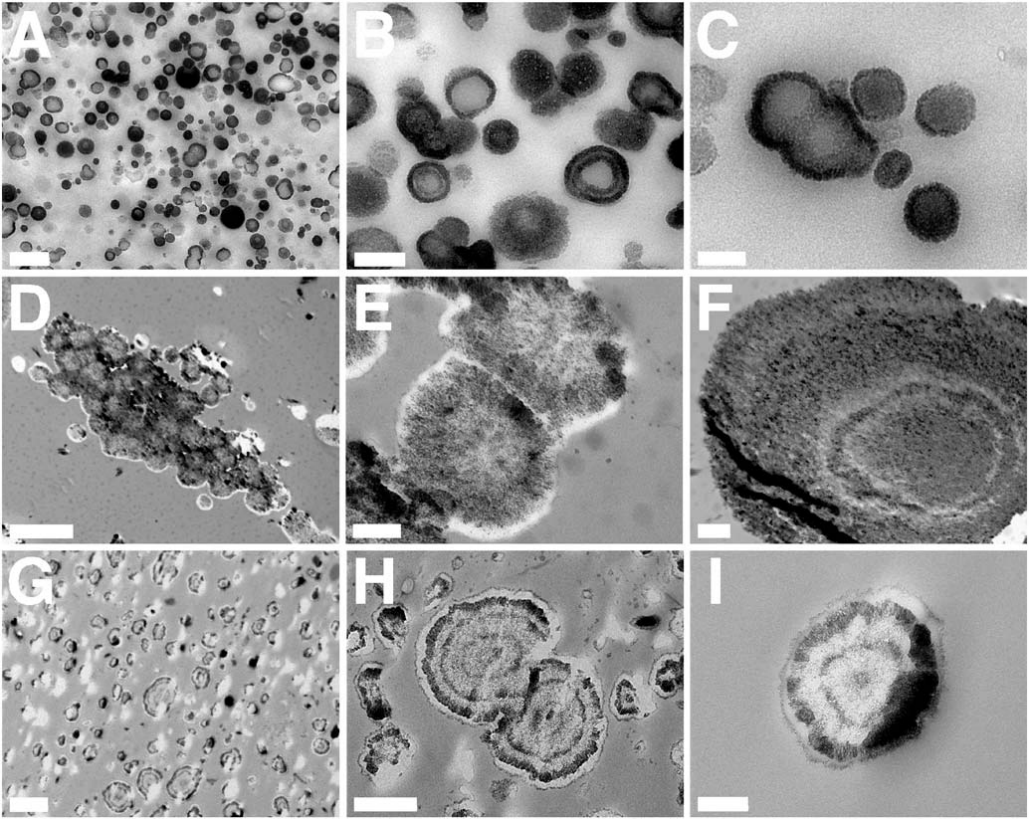
NLP and NB seen by thin-section TEM. (A–C) Calcium phosphate NLP produced as in Fig. 3 and incubated overnight in DMEM. (A) shows heterogenous particles, while (B and C) represent enlarged views of (A), depicting round particles with thick concentric wall layers and crystalline surface with needle-like projections. Some particles appear to be undergoing cell division. (D–F) Calcium carbonate NLP obtained from the inoculation of 10 mM of CaCl_2_ and (NH_4_)_2_CO_3_ into DMEM, followed by overnight incubation in serum-free DMEM. (D) Aggregation of NLP to produce coalesced particles. (E and F) Enlarged images of (D), showing cell-division forms (E) and concentric multi-layers (F) with dense coating and crystalline structure. (E) Higher magnification image of the same NLP shown in D depicts a cellular division-like formation. Note the crystalline appearance of their surface and the interior of the particles. (G–I) “Nanons” after 2 days in serum-free DMEM, revealing multilayer crystalline rings surrounding an electron dense core. Scale bars: 100 nm (C); 200 nm (B, I); 500 nm (A, E, H); 1 µm (F, G); 5 µm (D).

Under thin-section electron microscopy, NLP and NB also displayed virtually identical morphologies (compare Fig. 8A–F showing NLP with Fig. 8G–I for “nanons” as well as pictures shown for NB in refs. 1 and 2). Both NLP and the various NB showed characteristic thick walls, sometimes with multiple layers, and either smooth surfaces or outward projecting spindles. Particles were also seen to coalesce into larger lamellar structures (Fig. 8D and E).

### Role of carbonate and magnesium in the formation of NLP

The presence of either excess carbonate (2–8× phosphate concentrations), Mg^2+^, or a combination of both, in the initial precipitation mixture led to a predominance of small amorphous NLP instead of biofilms (Fig. 7G and H). By visual inspection, the NLP pellets formed in the presence of high amounts of carbonate or Mg^2+^ were not only smaller in volume, decreasing in inverse proportion to an increase in the concentration of either carbonate or Mg^2+^, but in the case of excess carbonate, they also looked white-chalky and were easier to disperse. In contrast, NLP formed with low amounts of either carbonate or Mg^2+^ were not only more voluminous but also more gelatinous, sticky, and grayish in appearance, and were much harder to disperse. The effects of carbonate and Mg^2+^ were clearly additive. The same morphological and visual impressions seen with high levels of input carbonate also correlated with higher Ca∶P ratios, as revealed by EDX analysis, with Ca∶P ratio reaching 2∶1 or higher ratios when NLP were formed with carbonate:phosphate ratios of 8∶1 (data not shown). In the case of both excess carbonate and Mg^2+^, FTIR and Raman spectroscopies also revealed higher carbonate peaks with a much slower rise for the phosphate peak. XRD analysis demonstrated a slower accumulation of HAP under these same conditions, with the initial predominance of amorphous patterns and mono-calcium phosphate peaks that converted much more slowly to HAP crystals (not shown). The influence of carbonate and Mg^2+^ on NLP formation may be physiologically relevant given the high concentrations of these ions in the culture medium. For example, as noted earlier for DMEM, carbonate at 44 mM exceeds Ca^2+^ (1.8 mM) by 24 times and phosphate (0.9 mM) by 49 times. At such high carbonate:phosphate ratios, it would appear that carbonate should inhibit HAP accumulation and biofilm formation while favoring the formation of small, coccoid-like amorphous calcium particles which nonetheless should still be expected to slowly accumulate HAP and gradually convert into biofilms. That is, while HAP inhibitors like carbonate or Mg^2+^ are seen to delay the accumulation of HAP by NLP in culture medium, the much higher binding affinity known to exist between calcium and phosphate, as compared with calcium and carbonate, should still favor nevertheless the irreversible formation of HAP over time.

### Inhibitory effect of serum on NLP formation

**Figure 9:**
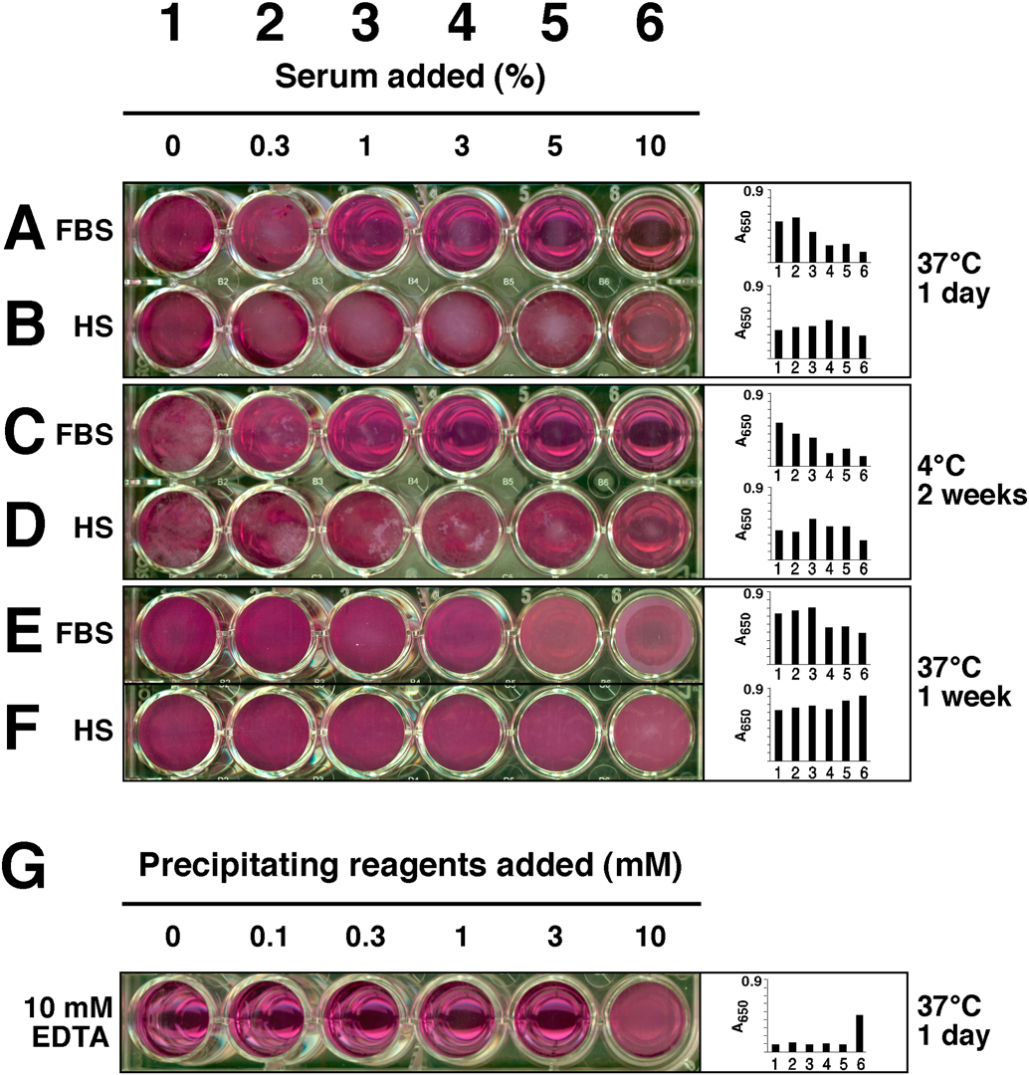
Effect of serum, temperature, and EDTA on NLP formation. NLP were prepared from 3 mM of CaCl_2_, Na_2_CO_3_, and NaH_2_PO_4_ in DMEM containing different amounts of FBS or HS, as indicated, followed by incubation at 37°C for 1 day (A and B), 1 week (E and F), or 4°C for 2 weeks (C and D). Note that FBS had a stronger, dose-dependent inhibitory effect on NLP formation in all 3 situations when compared with HS. Inhibition could be seen for 2 weeks at 4°C, but at 37°C, it was overcome after 1 week, and this release of inhibition was more evident with HS. (G) NLP were prepared exactly as in Fig. 1B, by adding solutions of 0.25 M CaCl_2_, Na_2_CO_3_, and NaH_2_PO_4_ (pH 7.4) to DMEM to the indicated concentrations in the presence of 10 mM EDTA, followed by incubation for 1 day. Addition of EDTA into the precipitation mix decreased the amount of NLP, except for well 6, containing 10 mM of precipitating reagents, indicating that excess EDTA is needed for the inhibition to work.

Having established that NLP and NB are morphologically and chemically equivalent, the earlier unexpected, serum-mediated inhibition on NB formation prompted us to revisit with this phenomenon by studying the role of serum in NLP formation, since the latter is more amenable to biochemical characterization than the elusive NB phenomenology. The effects of serum on NLP formation could be analyzed in a similar manner, as done initially with NB, using different amounts as well as sources of serum. Thus, under the conditions studied, FBS and HS were shown to support NLP formation in low amounts (up to 1% vol.∶vol.). However, as seen earlier for NB, serum was found to inhibit NLP formation at higher amounts, and this inhibitory effect was much more pronounced with FBS than with HS, as demonstrated by the dose-dependence experiments shown in Figure 9A and B. At 1% or more FBS, inhibition of NLP formation could be seen with 1 mM each of calcium, carbonate and phosphate levels added, while HS inhibited NLP formation at 5% or more (Fig. 9B shows inhibitory effect only up to 10% serum but similar inhibition was seen with higher serum concentrations). The amount of inhibition depended on the exogenous amounts of calcium, carbonate, and phosphate added, with higher amounts of these reagents clearly overriding the inhibitory effects of serum. Thus, when 10 mM each of calcium, carbonate, and phosphate was present, 3% or more FBS and 10% HS were needed to exert similar inhibitory effects (not shown). Conversely, as shown earlier, when 1% of FBS was maintained constant throughout the precipitation experiment, this same inhibition could be overridden with 3 mM each of calcium, carbonate, and phosphate (Fig. 1B, 2^nd^ row, labeled “1% FBS”). The extent of serum-mediated inhibition varied with the lot of serum used, with large margins of error seen among the serum batches tested.

When the same 24 well plate was subsequently stored at 4°C, the inhibition on NLP formation seen with high doses of either FBS or HS persisted and was still evident 2 weeks after the initial seeding (Fig. 9C and D). At higher temperatures like room temperature or 37°C, however, this inhibition turned out to be transient, and after 1 week of incubation at 37°C, precipitation of NLP became noticeable even in the wells containing as high as 30% FBS or HS (Fig. 9E and F). The initial inhibition period varied also as a function of the input levels of calcium, carbonate and phosphate, and inhibition was prolonged for a longer period of time with lower levels of calcium, carbonate and phosphate inoculated. At 3 mM each of calcium, carbonate, and phosphate, the initial inhibition seen with various doses of FBS could be sustained for at least 3 days, whereas the inhibition seen with its HS counterpart was completely overcome after this incubation. Likewise, at 10 mM each of calcium, carbonate, and phosphate, the initial inhibition seen with both FBS and HS was seen to be overridden after 2 days, even with the higher doses of FBS and HS used (not shown). Longer periods of inhibition were also observed with incubation done at room temperature. In general, by two weeks of incubation at room temperature, the inhibitory effect produced by serum was completely overcome irrespective of the amount of FBS or HS used (up to 30%; data not shown). However, the NLP precipitates formed at widely different doses of serum appeared different to visual inspection. Thus, NLP formed in the presence of 5% or more of FBS appeared finer and more granular in texture, while NLP formed in the absence of serum or at 1% or lower amounts of FBS appeared coarse and clumpy. On the other hand, these same differences were not as obvious with different amounts of HS.

NLP formation seen both in the presence and absence of serum was dependent on calcium, as shown by the inhibition produced with either EDTA (Fig. 9G) or EGTA (not shown), or by using DMEM containing no calcium (also not shown). On the other hand, DMEM without phosphate still produced NLP precipitates but these were significantly smaller in volume as compared to those seen with control medium (not shown).

**Figure 10:**
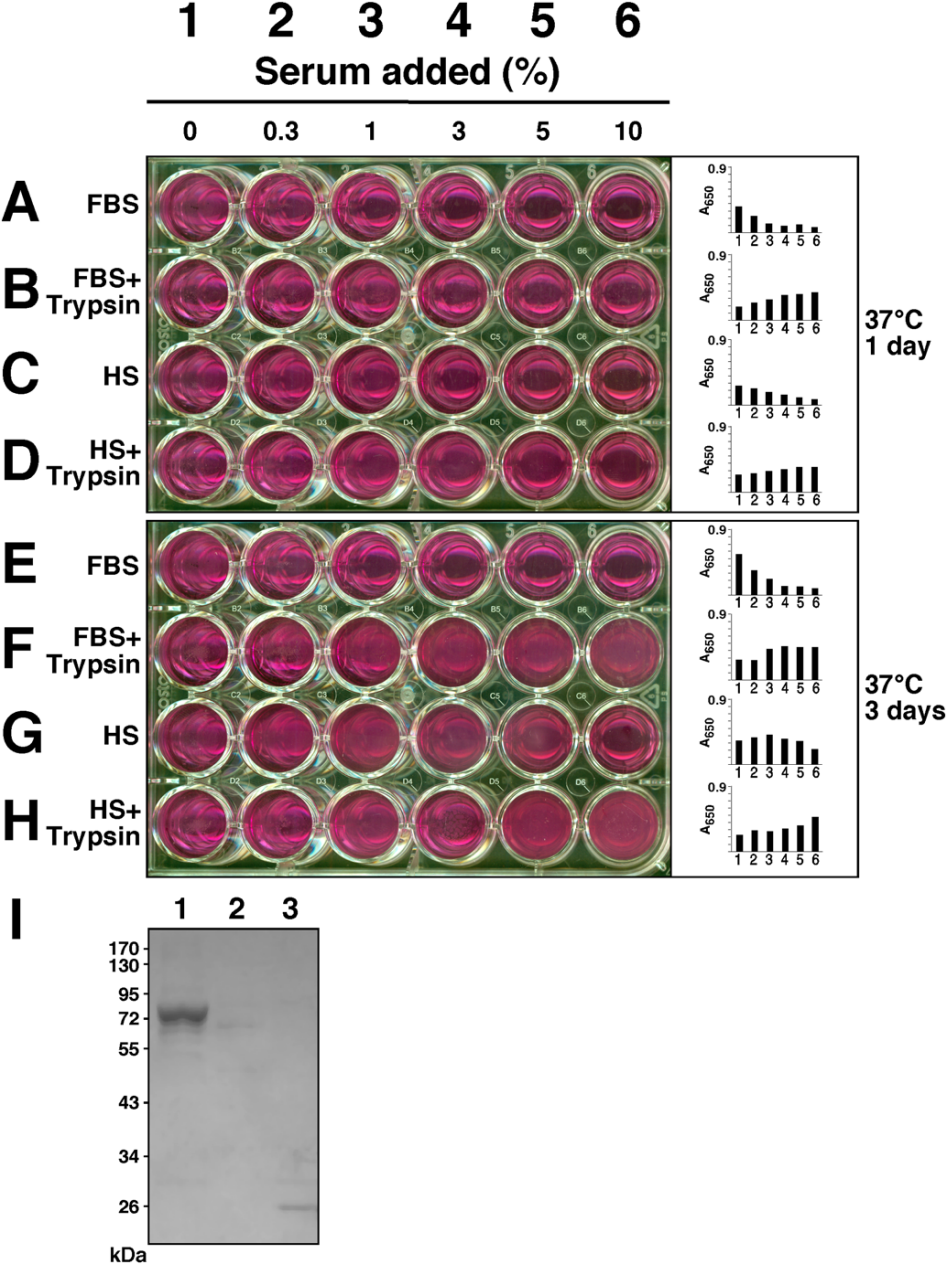
Effect of trypsin on NLP formation. NLP were prepared by adding 1 mM of CaCl_2_, Na_2_CO_3_, and NaH_2_PO_4_ to DMEM containing the indicated amounts of serum, in the presence or absence of 0.5% trypsin, followed by incubation at 37°C for 1 day (A–D) or 3 days (E–H). Trypsin was shown not only to release the inhibition caused by FBS or HS, but also to produce a dose-dependent increase in the amount of precipitation that increased with the serum dosage. (I) Trypsin treatment on NLP as demonstrated by SDS-PAGE. NLP formed as in Fig. 9 from 3 mM each of the 3 precipitating reagents as well as 1% FBS in DMEM, either in the absence (lane 1) or presence (lane 2) of 0.5% trypsin. After overnight incubation of the 1 ml incubation mixture at 37°C, 100 µl was removed, pelleted by centrifugation, and washed twice in DMEM, and resuspended in 16 µl of 50 mM EDTA in double-distilled water for SDS-PAGE. Lane 3 contains 5 µg of trypsin, as control, which shows as a 25 kDa band. Note a prominent 72 kDa band and two fainter 54 kDa and 30 kDa bands associated with FBS NLP (lane 1), all of which disappears with trypsin treatment (lane 2).

The inhibitory effects of serum on NLP formation could be overcome in the presence of trypsin. Figure 10A and C show the serum-dependent inhibition on NLP precipitation produced by 1 mM each of calcium, carbonate, and phosphate inoculated into DMEM, followed by overnight incubation at 37°C. As expected from earlier experiments, increasing amounts of FBS (Fig. 10A) or HS (Fig. 10C) were seen to inhibit NLP formation. Treatment with 0.5% trypsin under these conditions not only removed the serum-mediated inhibition but appeared to facilitate NLP formation as a function of the amount of serum added, with more serum inducing more precipitation (Fig. 10B and D). That is, rather than being inhibitory, both FBS and HS added in the presence of trypsin now induced a dose-dependent accumulation of NLP. As an additional control, the initial serum-induced inhibition on NLP formation could be seen to be sustained for at least 3 days in the absence of trypsin (Fig. 10E and G), an inhibition that was again more noticeable with FBS compared to HS. By comparison, the trypsin effect was stable, resulting in steady accumulation of NLP with incubation time (Fig. 10F and H).

To ascertain the efficacy of the trypsin treatment here in effecting proteolytic digestion, sodium dodecyl sulfate-polyacrylamide gel electrophoresis (SDS-PAGE) was used to analyze trypsin-treated NLP formed in the presence of FBS. Figure 10I shows the protein profile of FBS NLP that had been treated with 0.5% trypsin (Fig. 10I, lane 2) compared to that of control FBS NLP formed without trypsin treatment (Fig. 10I, lane 1). All three protein bands associated with FBS NLP (a prominent 72 kDa band, and two fainter bands of 54 kDa and 30 kDa) disappeared under our trypsin treatment conditions. The proteins associated with both NB and NLP will be discussed in greater detail later in this study. As a further control, trypsin alone was shown as a 25 kDa protein, unrelated to any of the three bands shown here (Fig. 10I, lane 3). These results clearly indicate that factors which inhibit calcium or apatite crystallization are present in both sera, but that higher amounts are present in FBS compared to HS. These factors initially inhibit the formation of NLP, but this inhibition can be overcome slowly by means of a spontaneous and temperature-dependent process or much more rapidly by trypsin treatment. These observations suggest that the same trypsin-sensitive inhibitory factors may be proteinaceous in nature. Once released of this trypsin-sensitive inhibition, both FBS and HS are now seen to enhance the formation of NLP in a serum dose-dependent manner, implying that non-proteinaceous factors may be present in both FBS and HS that support the formation of NLP, but these become effective only when the initial trypsin-sensitive inhibitory step has been overcome.

Once formed, these same NLP could be transferred to new culture medium by serial passage, similar to what had been reported earlier for NB [1], [2]. NLP formed in the presence or absence of serum could be transferred to either serum-free or serum-containing medium, or alternately. However, in our hands, the serial passage of NLP through serum-free DMEM resulted in the progressive decrease of precipitates in contrast to that seen with serum-containing DMEM. Likewise, the inoculation of NLP to DMEM with high amounts of serum (10% or more) also produced stagnation of NLP growth, which reverted only after several weeks of incubation. This slow growth of NLP could be accelerated by inoculating NLP into DMEM containing lower amounts of FBS. As detailed later, by subjecting NLP to passage through various kinds of culture conditions, we found that the chemical composition of NLP could also be modulated.

This inhibitory effect of serum, being more pronounced with FBS, stands in contrast against the bulk of NB literature that had previously used both FBS and HS not only as sources of NB but also as sources of nutrients to support NB proliferation [1]–[6]. We noticed that at least one earlier report which failed to demonstrate NB in culture had also used FBS at 10% [14] while Cisar et al. [12] had observed that NB obtained from saliva samples grew much faster in serum-free conditions than in the presence of 10% γ-FBS. The latter authors also found that NB could be obtained more readily from saliva than from FBS [12]. Significantly, we came across one recent review by the discoverers of NB in which they described mineralization of NB only with FBS at concentrations of 5% or less, in line with the results presented here, but no reference to any primary data was given in that review [4]. Another more recent publication by the same authors also briefly cited increased calcification of CNP seen with FBS under 5%, but the same authors failed to conceptualize the inhibitory notion described here and they also did not attempt to reconcile this observation with the contradictory fact that serum had been used as the primary source for NB (or CNP) that led to their discovery in the first place [49].

Taken together, our results indicate that both seeding and inhibitory factors for NB may be present in the serum. At 10% serum, an inoculum size used by all the earlier reports to demonstrate NB propagation from serum, inhibition on NB growth appeared to predominate. This inhibition was shown to be transient and temperature-dependent, with inhibition being overcome faster with further incubation at 37°C, but not at 4°C, suggesting that a temperature-sensitive conversion step may be required for the propagation of NB.

Moreover, it is now apparent that all previous published data relying on the inoculation of various NB seeding factors from the specimens being tested (protein homogenates, serum factors) could further modulate the inhibition parameters exemplified here, adding ambiguity to any interpretation concerning the origin of NB.

### Proteins associated with NLP and NB studied by SDS-PAGE

To date, little published information can be found concerning the protein profiles associated with NB, and when available, they always appear as protein smears [4], [16], [17]. Cisar et al. [12] reported earlier that human saliva cultured in serum also produced NB yielding protein smears when analyzed by SDS-PAGE. However, under serum-free conditions, the same saliva samples produced NB with a simple protein profile consisting of only a few protein bands, an observation deemed incompatible with the protein profile of living microorganisms, expected to be more complex. Based on these and other observations, the same authors concluded that their NB are lifeless entities consisting of saliva proteins that bind avidly to apatite [12]. Recently, Raoult et al. [19] have shown that *Nanobacteria* sp. (“nanon”) particles cultured in FBS that were subsequently passaged through serum-free DMEM also produced a simple profile of no more than three major proteins, one of which (65 kDa) was identified as fetuin-A. Our own earlier study [20] indicated that human NB and NLP derived from human serum reacted with NB-specific monoclonal antibodies that in turn reacted strongly with albumin.

**Figure 11:**
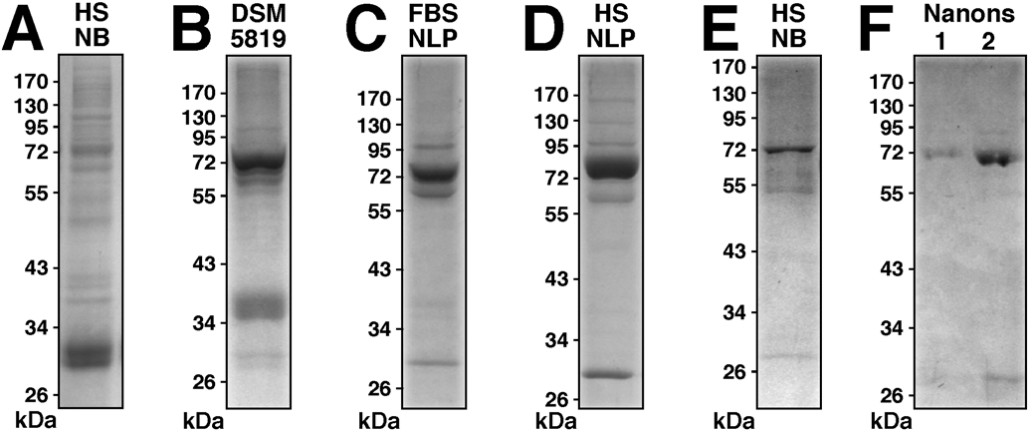
Protein profiles of NLP and NB cultured from serum. (A) Protein profiles of (A) NB cultured from HS and DMEM, as in Fig. 3; (B) NB strain DSM 5819 subcultured in serum-free DMEM for 2 days; (C) FBS NLP prepared as in Fig. 3 in the presence of 5% FBS; (D) HS NLP prepared as in Fig. 3 in the presence of 5% HS. (E) NB as in (A) after three 2-day serial passages through serum-free DMEM. (F) Lane 1: “Nanons” after two 2-day passages in serum-free DMEM. Lane 2: “Nanons” after two 2-day passages in DMEM containing 10% FBS. Gels were stained with Coomassie blue. The protein samples loaded onto each lane and their preparation are described in the *Materials and Methods*.

Here, we sought to examine more carefully the protein composition of NLP/NB with an eye to better understanding their formation. Human NB were prepared according to published protocols in the presence of 10% HS, as outlined in the *Materials and Methods*. NB analyzed by SDS-PAGE and stained by Coomassie blue showed a similar smearing of proteins, obtained after extensive washes with buffers containing physiological amounts of salts, ruling out weak electrostatic associations (Fig. 11A). This protein profile was found to be comparable to that obtained with the three DSM strains of NB. Upon SDS-PAGE, this NB material displayed a similar multiplicity of bands which was identical for all three specimens obtained (DSM 5819 profile shown in Fig. 11B). In addition to a prominence of protein bands in the 55–75 kDa range, we also noticed the presence of an additional band of 35–38 kDa present occasionally in the protein profiles of DSM strains (Fig. 11B). Since this profile was also obtained after several washes with high ionic strength buffers followed by prolonged dissolution using 50 mM EDTA, it appears to be representative of this particular strain of NB.

The profiles presented here for NB differed markedly from those seen for NLP obtained by inoculating calcium-carbonate-phosphate ions into DMEM in the presence of serum. We prepared NLP in DMEM containing 5% FBS or HS, followed by washing them twice with a high-ionic strength buffer and EDTA dissolution. The protein profiles obtained for the NLP prepared in FBS and HS are shown in Figure 11C and D. Both profiles essentially consisted of three major bands of molecular weight of about 70 kDa (ranging from 66 to 75 kDa), 60 kDa (range of 52–65 kDa), and 30 kDa (range of 27–33 kDa). We noticed that the position of the middle band varied significantly with the lot of serum used, sometimes associating with sub-bands, and it was generally lower in the case of human material when compared with its bovine counterpart.

We reasoned that the discrepancies seen between the various preparations of NLP or NB could be due to the differences in the serum input and incubation conditions associated with the various methodologies used to generate NLP/NB. In the case of Raoult et al.'s study [19], the three prominent bands were only obtained after diluting “nanons” in serum-free medium and using silver nitrate for staining, compared to the less sensitive Coomassie blue stain used here. To address this issue, we incubated NB obtained from HS in serum-free DMEM at 37°C. The precipitate collected after 3 days in serum-free conditions was then washed in DMEM and subjected to SDS-PAGE analysis as before. As shown in Figure 11E, only the three main bands of 66–75 kDa, 52–65 kDa, and 27–33 kDa were again prominently seen (contrast this profile with Fig. 11A).

It should be noted that these three bands, obtained for both bovine and human NLP, are virtually indistinguishable from the gel profile published by Raoult et al. [19] for “nanons” grown initially in FBS and maintained in serum-free conditions. In our hands, the so-called “nanons” maintained in serum free conditions for 5 days gave only a weak band at 70 kDa and two faint bands at 60 kDa and 30 kDa when stained by Coomassie blue (Fig. 11F, lane 1). However, when the same samples were passaged twice through DMEM containing 10% FBS, the two bands of 70 kDa and 30 kDa gained prominence while the 60 kDa band remained barely visible (Fig. 11F, lane 2). When next passaged through serum-free conditions, the same bands again became progressively fainter with time until they disappeared (not shown). It appears that the passage of “nanons” through serum-free conditions done earlier by Raoult et al. [19] and repeated here exerted not only a selection for more strongly-binding proteins but also a diluting effect on serum proteins that adhere to NB.

**Figure 12:**
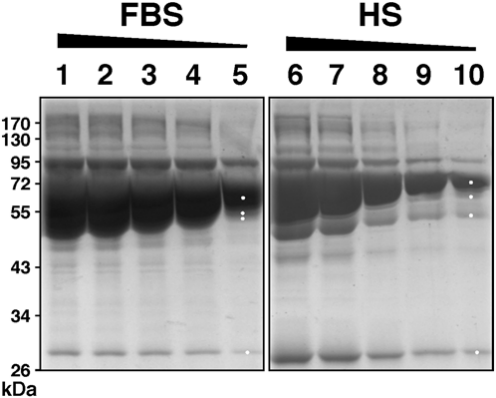
Protein profiles of whole FBS and HS. SDS-PAGE of decreasing amounts of whole FBS (lanes 1–5), and HS (lanes 6–10). FBS or HS was used at 1∶100 dilution, with the following amounts of protein loaded onto each lane: lanes 1–5: 4 µg, 3.5 µg, 3 µg, 2.5 µg, and 1.5 µg, respectively; and lanes 6–10: 3 µg, 1.8 µg, 1.2 µg, 0.6 µg, and 0.3 µg, respectively. The white dots shown in lanes 5 and 10 correspond to the bands excised for protein identification by MALDI-TOF mass fingerprint analysis.

To confirm this notion, we analyzed next the protein profiles of whole sera that were used for the preparation of NLP and NB. Both whole FBS and HS were found to be enriched for the exact three major protein bands associated with NLP, and their predominant presence could be more easily assessed through a dose-dependent dilution of the kind shown in Figure 12. As detailed through protein sequencing shown in the next section, the three major proteins associated with serum turned out to be identical to the exact three proteins associated with both NLP and NB.

These results indicate that the three calcium or apatite-binding proteins seen associated with NLP and NB are also the predominant proteins present in the serum that are probably being adhered and selected by NLP/NB at the expense of other minor protein species. It is clear however that with continued passage in serum-free conditions, even the three predominant protein bands will eventually disappear or remain barely visible by Coomassie blue staining, which may explain why Raoult et al. [19] had to use a much more sensitive dye (silver nitrate) to visualize these same protein bands in serum-free conditions. Furthermore, the protein smear found associated with NB, accompanied by the fading of the three prominent bands for example, could represent the result of proteolytic breakdown of serum proteins following prolonged incubation at 37°C. Together, these data clarify an important aspect of the NB phenomenology, namely the possibility that the NB protein coating may be derived from direct calcium or apatite binding to common serum proteins that seemingly are also among the more abundant proteins in the serum.

**Figure 13:**
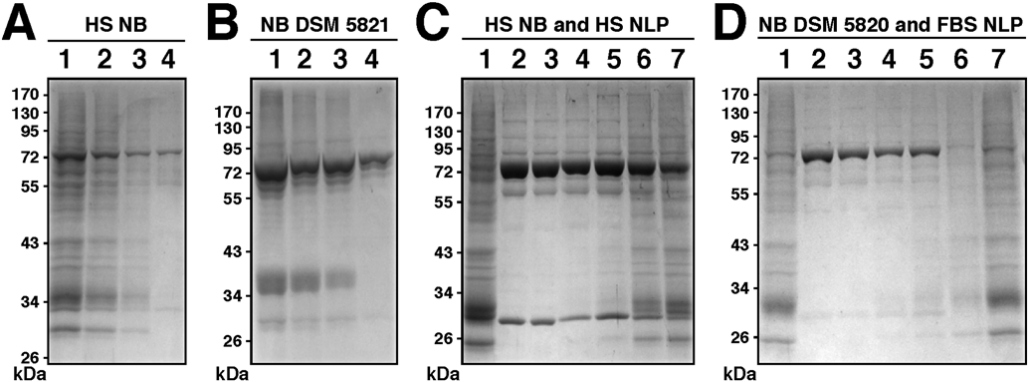
Protein profiles of NLP and NB as determined by their passage through serum-free or serum-containing medium. (A) Protein profile of HS NB maintained in DMEM containing 5% HS (lane 1) followed by transfer to serum-free DMEM and incubation for 1 day (lane 2), 4 days (lane 3), and 6 days (lane 4), after which proteins were analyzed by SDS-PAGE. A gradual disappearance of proteins bands is seen with increased incubation time in serum-free medium. (B) Protein profile of NB strain DSM 5821 after 1 day in DMEM containing 2% FBS (lane 1), followed by transfer to serum-free DMEM for 1 day (lane 2), 4 days (lane 3), and 6 days (lane 4), again showing a gradual disappearance of bands. (C) Protein profiles of NB obtained from 10% HS as in Fig. 3 (lane 1) and HS NLP formed as in Fig. 3 except that 5% HS was present in the precipitating mixture (lane 2). HS NLP were then transferred to DMEM containing 5% HS and incubated for the following periods of time: 1 hr (lane 3), 1 day (lane 4), 4 days (lane 5), 10 days (lane 6), and 14 days (lane 7). A gradual increase of bands of low molecular weight could be seen along with a fading of the high molecular weight bands, especially the 70–72 kDa band. (D) Protein profiles of NB strain DSM 5820 maintained in 10% γ-irradiated FBS (lane 1) and FBS NLP obtained as in Fig. 3, except that 5% FBS was added to the precipitating mixture (lane 2). FBS NLP were inoculated into DMEM containing 5% FBS and incubated for the following periods of time: 2 hr (lane 3), 1 day (lane 4), 4 days (lane 5), 10 days (lane 6), and 14 days (lane 7). By day 14, the protein profile of these FBS NLP, with an increase in the number of bands and a loss of the 70–72 kDa band, closely resembled that of DSM 5820 (lane 1).

Since serum has been routinely used both as a source progenitor as well as a nutrient basis for NB, we next sought to study further the influence of the serum milieu on the protein profiles of both NLP and NB. We reasoned that the protein coating of NLP/NB should mirror their passage history with regards to the presence or absence of serum. Figure 13A shows the protein gel profile of human NB maintained in 5% HS (lane 1) that had been inoculated into serum-free DMEM (lanes 2–4). The gradual disappearance of protein bands was obvious over a course of several days, with the three more predominant bands remaining by the end of day 6 (lane 4). The same phenomenon could be seen with the DSM 5821 strain, cultured in 2% FBS, that was subsequently inoculated into serum-free FBS (Fig. 13B). Again, over a period of several days, only a few major bands remained (contrast lane 4 obtained on day 6 of incubation with lane 1, obtained for DSM 5821 particles maintained in 2% FBS). Conversely, similar passage histories could be established with human NLP obtained with exogenous calcium-carbonate-phosphate ions (Fig. 13C). Here, human NLP showing only three major bands (Fig. 13C, lane 2) were first contrasted with the smear of bands seen with NB obtained by inoculation of 10% HS into DMEM using published protocols as before (Fig. 13C, lane 1). Figure 13C shows that these same NLP could be made to acquire many other protein bands when inoculated into DMEM containing 10% HS (lanes 3–7). By day 10, there was a large increase of lower molecular weight bands accompanied by a decrease of the 70 kDa (Fig. 13C, lane 6). After 14 days of incubation in the presence of serum, the protein profile seen associated with the precipitating particles (Fig. 13C, lane 7) closely resembled the protein profile of NB (lane 1). Similar results were obtained with NLP formed in the absence of serum and that had been inoculated subsequently into DMEM containing 5% FBS (Fig. 13D, lanes 2–7). There was a gradual smearing of the protein bands, with a loss of the 70 kDa band and an increase of low molecular weight bands (see Fig. 13D, lanes 6 and 7, corresponding respectively to days 10 and 14 of incubation in the presence of 5% FBS). Again, this profile was virtually indistinguishable from that obtained for DSM 5820 maintained in 10% FBS (Fig. 13D, lane 1).

Using this approach, that is, by simply interchanging culture serum conditions, we were able to obtain protein profiles for NB and NLP that were virtually identical to each other. Even the serum species (bovine versus human) could be interchanged resulting in final profiles that were virtually indistinguishable from native configurations (not shown). Since the numerous previous NB studies relied largely on inoculating human tissue homogenates or body fluids into FBS-containing medium, this same interchange of species may very well have happened at the level of NB protein coating. Since all protein gel profiles here were obtained after repeated washes in high ionic strength buffers, we conclude that these complex protein profiles may represent the true NB composition. On the other hand, the marked reduction of the number of bands associated with NB seen under serum-free conditions, observed here as well as in earlier studies [12], [19], can be explained by a dilution effect, with the initial serum proteins bound to NB becoming diluted by new mineral complexes being formed, eventually ending with just the more abundant proteins remaining visible. Upon being reinoculated back into serum-containing medium, new serum proteins adhere to the NB complexes, thereby starting a new cycle with respect to protein composition. This alternative view further implies that NB are not only lifeless entities but their protein coating is a direct result of binding to proteins found in the surrounding milieu.

It is not clear why the various NB specimens or strains studied show variability in their protein profile when analyzed by SDS-PAGE. We have noticed that in the case of “nanons,” the number of protein bands is significantly fewer than that seen with NB or with the DSM 5819–5821 strains. That is, while NB and the DSM strains tend to show protein smears on SDS-gels (Fig. 11A and B, respectively), “nanons” that have been maintained in DMEM in the presence of FBS show a simple profile consisting of a few major bands (Fig. 11F, lane 2). However, based on chemical and morphological analyses, “nanons” and the other NB specimens and strains do not display any noticeable differences. More experiments will be needed to address the protein profile differences seen between the various NB strains obtained to date.

### Identification of the proteins associated with NLP and NB

**Figure 14:**
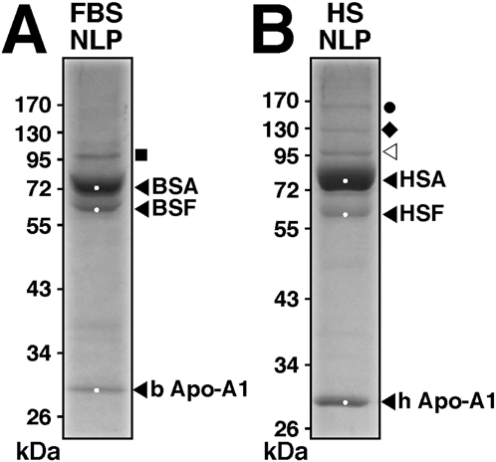
Protein identification in NLP prepared from FBS and HS. Protein profiles correspond to Figs. 11C and D, reproduced here to illustrate the protein identification procedure used as well as the results obtained. NLP were prepared from DMEM containing 5% FBS or 5% HS that had been precipitated with 10 mM each of CaCl_2_, Na_2_CO_3_, and NaH_2_PO_4_. Following SDS-PAGE and Coomassie blue staining, the bands were excised (white dots) and submitted to identification by MALDI-TOF mass fingerprint analysis. The proteins identified are given along with their positions. Abbreviations used: BSA, bovine serum albumin; BSF, bovine serum fetuin-A; b Apo-A1, bovine apolipoprotein A1; HSA, human serum albumin; HSF, human serum fetuin-A; and h Apo-A1, human apolipoprotein A1. Other minor protein bands include a bovine 95 kDa band (▪) which was identified as bovine serotransferrin precursor (2 out of 2 trials, or 2/2), and other minor human bands at 165 kDa (•), 130 kDa (♦), 95 kDa (◃) identified as human complement C3 (2/2), human antithrombin (2/2), and human complement C3 (2/2), respectively.

The major bands from the protein gels (Fig. 11) were excised for protein identification. As an example, the gels in Figure 11C and D were relabeled as Figure 14A and B, now showing the exact excision positions, seen as white dots on the protein bands. The protein materials were then in-gel digested with trypsin, and submitted to identification by MALDI-TOF mass fingerprint analysis and comparison with existing database. The proteins identified and that fulfilled standard criteria for identification (see *Materials and Methods*) are listed in Table 1. The identity of the proteins obtained by MALDI-TOF mass fingerprint analysis was confirmed by tandem mass spectrometry (MS-MS) analysis. The amino acid sequences of the peptide peaks that have been selected for the confirmation of the proteins most commonly identified in this study are shown in Table 1. The major protein from each NLP profile had a molecular weight of 66–75 kDa (Fig. 14A and B). This protein was identified by MALDI-TOF mass spectrometry as bovine serum albumin (BSA) in FBS-derived NLP (Fig. 14A, Table 1) and as HSA in HS-derived NLP (Fig. 14B, Table 1). These results confirm our earlier Western blot observations where HSA was found to bind NB cultured from HS, as identified using NB-specific antibodies [20]. In our hands, albumin was the most commonly identified protein in the 66–75 kDa band (see Table 1), not only for human NB (2 times out of a total of 3 trials) and NLP (6/6), but also for bovine NLP (5/6). Given that albumin is more abundant than any other proteins seen with both bovine and human NLP/NB, it is not clear why Raoult et al. [19] did not detect albumin in their study.

**Table 1:** NLP-binding proteins identified by MALDI-TOF mass spectrometry.

Protein Name^a^	Score^b^	Frequency^c^	Peptide Sequences Identified^d^
Bovine serum albumin	271	5/6	LGEYGFQNALIVR; DAFLGSFLYEYSR
Bovine fetuin-A	69	14/35	QDGQFSVLFTK; TPIVGQPSIPGGPVR
Bovine apolipoprotein A1	276	8/9	VAPLGEEFR; WHEEVEIYR
Human serum albumin	390	6/6	YLYEIAR; RHPDYSVVLLLR
Human fetuin-A	71	4/15	HTLNQIDEVK; EHAVEGDCDFQLLK
Human apolipoprotein A1	325	2/3	WQEEMELYR; THLAPYSDELR

a Corresponding gel profiles are shown in Fig. 14.

b Highest mass fingerprint score of the proteins identified by MASCOT search.

c Frequency of proteins obtained from distinct SDS-PAGE experiments as a function of the number of trials done.

d Peptide sequences of two selected peaks obtained by tandem mass spectrometry analysis.

Using a similar approach, the 52–65 kDa bands associated with the FBS and HS NLP were identified as bovine serum fetuin-A (BSF) and human serum fetuin-A (HSF), respectively (Fig. 14A and B; Table 1). However, to our initial surprise, and in contrast to the earlier findings of Raoult et al. [19] implying fetuin-A as the major constituent of NB, fetuin-A was not found consistently in the various samples analyzed. In fact, we obtained fetuin-A in less than half of the trials (14/35) for FBS NLP, and in one fourth of the trials (4/15) for HS NLP (Table 1). Furthermore, in our hands, we were not able to detect fetuin-A in human NB obtained from human serum according to published protocols as described in the *Materials and Methods* (data not shown). Other proteins such as albumin (16 out 50 combined trials for both bovine and human material), vitamin D-binding protein (4/50), anti-thrombin-III precursor (4/50), α1-anti-trypsin precursor (2/50), immunoglobulin-G chain C region (1/50), and immunoglobulin-M chain C region (1/50) were also obtained at this position. These observations suggest that the amount of HSF in this 52–65 kDa band is probably much lower than its bovine counterpart, and that other proteins may contribute to the intensity of the band seen in this position.

The third lower major bands of 27–33 kDa seen on the protein profiles of FBS and HS NLP (Fig. 14A and B) were identified as bovine apolipoprotein A1 (8/9) and human apolipoprotein A1 (2/3), respectively (see also Table 1). This result is consistent with Raoult et al.'s determination made on “nanons” grown in FBS [19]. In addition, the minor band of 95 kDa seen on the FBS NLP protein profiles (Fig. 14A; position marked by the full square symbol) was identified as serotransferrin precursor (2/2). On the other hand, the high molecular weight bands seen in HS NLP at the 95 kDa, 130 kDa, and 170 kDa (Fig. 14B, marked by the three other symbols), were identified respectively as human complement C3 (2/2), human antithrombin (2/2), and, again, as human complement C3 (2/2).

To verify whether these same proteins corresponded to the major bands seen with whole serum (Fig. 12), the three main bands from the serum protein gels were also excised and submitted to identification by MALDI-TOF mass fingerprint analysis (Fig. 12, lanes 5 and 10, marked by white dots). Albumin and apolipoprotein A1 were readily identified from both human and fetal bovine serum materials (1/1 each), but fetuin-A was not found in the 52–65 kDa band for either human or fetal bovine serum (2/2 each). Instead, interleukin-8 precursor (1/2) and BSA (1/2) were obtained at the 52–65 kDa position in whole FBS. For HS, we obtained α-1-syntrophin (1/2) and an uncharacterized protein KIAA0774 (1/2) at this position. Although it is likely that more sample trials would have identified fetuin-A, it is clear from these results that other proteins appear to contribute equally, if not more predominantly, to the intensity of this 52–65 kDa band. These results confirm the notion that the proteins found associated with NB largely reflect their predominance in the serum.

Several precautions were taken to ensure that the proteins associated with NLP were separated from the mineral phase prior to gel electrophoresis. Such precautions included treatment of NLP samples with 50 mM EDTA, 2.5% β-mercaptoethanol or 0.1 M DTT, and 2% SDS, followed by heating at 95°C for 5 minutes prior to electrophoresis. However, even under these conditions, the protein-mineral complexes were not completely dissolved as judged by the presence of small precipitates seen with subsequent centrifugation and by the retention of high molecular weight complexes visible in the space between the spacer and separating gels when the treated NLP samples subjected to SDS-PAGE analysis. In order to both circumvent this problem and have a more thorough determination of the entire protein repertoire associated with NLP, we also used in-solution trypsin digestion of untreated NLP samples followed by separation of the resulting peptides by reversed-phase liquid chromatography and their identification by tandem MS/MS analysis. Using this technique, several other high molecular weight proteins were found in addition to the three proteins mentioned earlier and displayed in Table 1 that were not readily visible by SDS-PAGE. These proteins included, in descending molecular weight, apolipoprotein B100 (516 kDa; 5/5 in HS NLP), apolipoprotein(a) (501 kDa; 1/5 in HS NLP), coagulation factor V (249 kDa; 4/13 in FBS NLP), complement component 4A (193 kDa; 5/5 in HS NLP), and α_2_-macroglobulin (163 kDa; 2/13 in FBS NLP; 5/5 in HS NLP). We noted the presence of other calcium-binding proteins like vitamin-D-binding protein (53 kDa; 12/13 in FBS NLP; 4/5 in HS NLP) and kininogen-1 (48 kDa; 5/5 in HS NLP). Fibronectin (257 kDa; 1/13 in FBS NLP; 5/5 in HS NLP) and vitronectin (54 kDa; 5/5 in HS NLP), two human serum proteins deemed to be potent nucleators of HAP [50], which were also found associated with the mineral phase of NLP.

That multiple proteins should bind to carbonate HAP used in these experiments is consistent with the well-known protein-binding affinity displayed by both this mineral and calcium [51]. The presence of phosphate groups on HAP crystals allows for their binding to basic amino acids from various proteins. In fact, it is this broad affinity for proteins that has made HAP useful for protein purification in the past [51].

Earlier, Raoult categorized NB as “fetuin-mineralo complexes” [19]. In their study, fetuin-A was shown to be present in NB cultured from both FBS and renal stones. Based on our own results, albumin must also be considered a major component of NB/NLP. In fact, in our hands, of the proteins discernible by SDS-PAGE, albumin has turned out to be the most abundant protein species associated with NB/NLP, be them of human or fetal bovine origin. Not surprisingly, albumin is one of the major proteins found in both human and bovine serum, where it is known to be present at concentrations as high as 35–45 mg/ml in human serum [52] and 23 mg/ml in FBS [53]. On the other hand, fetuin-A, while abundant in FBS at 10–21 mg/ml [54], is found at levels of 0.7–0.8 mg/ml in the adult human serum [55], or at levels that are 14–26× lower than those found in FBS.

As noted earlier, we failed to find fetuin-A in human NB cultured directly from HS, in spite of repeated attempts to sequence the 52–65 kDa protein band from gels. Fetuin-A was however found in both human NLP freshly prepared using calcium-carbonate-phosphate precipitation in the presence of HS, as well as in FBS-derived NB and NLP. These discrepancies may simply be due to the differences in concentrations of fetuin-A and albumin found in FBS versus HS. Compared to albumin, fetuin-A may be more selectively enriched and concentrated by both NLP and NB. For one, fetuin-A is known to display much stronger binding affinity for calcium (calcium-binding constants of 0.95×10^−4^ M for BSF and 1.24×10^−4^ M for HSF) than albumin (7×10^−3^ M for BSA) [56]. Furthermore, fetuin-A displays unique apatite-binding sites [ref. 25]; [reviewed in ref. 27]. Given these unique binding properties and the high concentration of fetuin-A in FBS, it would make sense for fetuin-A to be enriched in FBS-derived NB over other proteins. In the case of human fetuin-A, however, since it is found in much lower levels in HS compared to FBS, it does not seem to correspond to a major protein species of human NB in spite of its known high affinity for calcium and apatite.

These findings support the notion that the presence of the three predominant serum proteins as well as other proteins in NB may be largely circumstantial in the sense that it mirrors their availability in the milieu in which NB are being formed. In support of this view, even though albumin binds more weakly to calcium than fetuin-A, it had previously been shown to account for at least half of the calcium binding capacity quantified for HS, mainly due to its high serum concentration [57]. More recently, Heiss et al. [58] observed a similar prominence of albumin over fetuin-A in the constitution of the so-called “secondary calciprotein particles” obtained from human ascites, in spite of the stronger calcium-binding affinity of fetuin-A.

It should be noted that there are many other proteins that have been previously shown to bind to calcium and apatite and that may interact with NB [38], [59]. In fact, our own findings here point to the presence of multiple calcium-binding proteins present in the NB and NLP scaffold. For the first time, however, our ability to dissect NB through the reconstitution experiments described here provides a conceptual framework that explains the origin and nature of the protein repertoire found associated with NB.

### NB and NLP associated with other body fluids and their protein identification

To assess whether the NB/NLP phenomenon dissected here is unique to serum-derived materials, we inoculated several other body fluids into DMEM in order to form NB. Samples of saliva, urine, ascites, cerebrospinal fluid, pleural effusion, and synovial fluid were centrifuged, membrane-filtered, and directly inoculated into serum-free DMEM at dilutions between 1∶10 and 1∶100. Saliva was obtained from normal volunteers whereas the other fluids were obtained from pathological specimens. All samples resulted in slow, dose-dependent precipitation of particles after an initial incubation of 3–8 days. NB formed in this way could be passaged serially through DMEM without serum for at least three cycles (not shown). The only fluid that gave negative or marginal NB formation was urine. To enhance NB formation with urine, samples were obtained from pathological specimens collected from patients showing various degrees of kidney damage and proteinuria. We noticed that a higher level of proteinuria corresponded in general with a higher propensity of the same urine samples to form NB precipitates when inoculated into DMEM.

**Figure 15:**
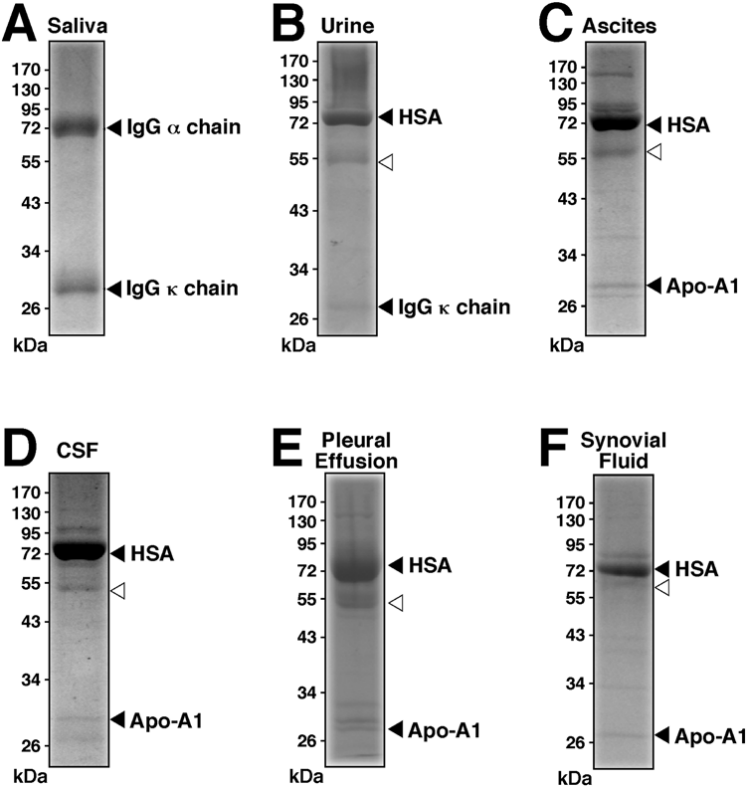
Proteins identified in NLP prepared from various body fluids. NLP were prepared from body fluids in the presence of CaCl_2_, Na_2_CO_3_, and NaH_2_PO_4_ added to final concentrations of 9 mM each for saliva, 7 mM for urine, and 8 mM for the other body fluids. Following SDS-PAGE, the NLP proteins identified by MALDI-TOF mass fingerprint analysis are shown next to each major band. Other minor bands in the 50–65 kDa range marked by the symbol (◃) include (B) IgG chain C region (obtained 1 out of 1 trial, or 1/1); (C) IgG chain C region (1/1); (D) HSA (1/3), IgG chain C region (1/3), and α-anti-trypsin (1/3); (E) HSA (2/2); and (F) vitamin-D binding protein (1/3), IgG chain C region (1/3), and Apo-A4 (1/3).

When examined by SEM and TEM, the NB formed from these fluids showed marked morphological similarity to the NB formed from serum (not shown). These same fluids also yielded large amounts of NLP when precipitated with a combination of calcium-carbonate-phosphate (*Materials and Methods*) that were virtually identical to the NB formed by their inoculation and slow incubation in DMEM. Figure 15 shows representative SDS-PAGE profiles for NLP obtained from the various body fluids mentioned earlier. After NLP formation, samples were washed as before with physiological buffers prior to gel electrophoresis. As seen for NLP derived from each fluid sample (Fig. 15A–F), relatively simple protein profiles were obtained, consisting of a few major bands, with the most prominent band being around 66–75 kDa. With the exception of the saliva sample, albumin was the predominant protein species found in this band for all the NLP studied (Fig. 15B–F). Apolipoprotein A1 was also present in all the fluid NLP studied except for saliva and urine (Fig. 15C–F).

It should be pointed out that fetuin-A was not found in any of our samples. Instead, for the 52–65 kDa position (marked by open triangles in Fig. 15B–F), the following proteins were identified: immunoglobulin-G chain C region (1/1) was found in the urine NLP; immunoglobulin-G chain C region (1/1) was found in the ascites NLP; HSA (1/3), immunoglobulin-G chain C region (1/3), and α1-anti-trypsin precursor (1/3) were found in the cerebrospinal fluid NLP; HSA (2/2) was found in the pleural effusion NLP; and vitamin-D binding protein (1/3), immunoglobulin-G chain C region (1/3), and apolipoprotein A4 (1/3) were found in the synovial fluid NLP.

For NLP derived from both saliva and urine, we found marked variability in the proteins identified. When NLP were prepared from the urine of patients with nephrotic syndrome (see *Materials and Methods*), HSA was found at the 66–75 kDa position in 2 out of 4 samples (Fig. 15B shows one of the two urine samples where HSA was obtained). In the two other urine NLP samples, Tamm-Horsfall urinary glycoprotein (1/8), complement C3 (1/8), immunoglobulin-A chain C region (1/8), immunoglobulin-G chain C region (1/8), α1-anti-trypsin precursor (1/8), and fibrinogen (1/8), were found instead. At the position indicated by the open triangle in Figure 15B, immunoglobulin-G chain C region was found in two urine NLP samples (4/4), but this band was absent in the two other urine NLP samples (data not shown). Immunoglobulin-K chain C region (1/3; shown in Fig 15B), glycogenin-1 (1/3), and serum amyloid-P component (1/3) were found at the 27–33 kDa position for urine NLP.

In the case of saliva NLP, HSA was not found at the 66–75 kDa position. Instead, immunoglobulin-A chain C region (4/8), human fukutin-related protein (2/8), immunoglobulin-K chain C region (1/8), and salivary amylase (1/8) were found at this position. Protein UNQ773 (2/4), immunoglobulin-K chain V region (1/4; shown in Fig. 15A), and novel nuclear protein-1 (1/4) were also found at the 27–33 kDa position for saliva NLP.

The results presented here suggest that both albumin and apolipoprotein A1 are major constituents of NB/NLP obtained from internal body fluids besides serum, whereas the body-excreted fluids saliva and urine show marked individual protein variability depending on the specimens collected. That fetuin-A could not be found in any one of the fluid samples studied here reinforces our contention that the protein make-up of NB may be circumstantial at best, and it may depend entirely on the availability of specific calcium-binding proteins in the surrounding milieu. In this sense, none of the calcium or apatite-binding proteins shown here or elsewhere can be deemed to be absolutely necessary for generating NB.

**Figure 16:**
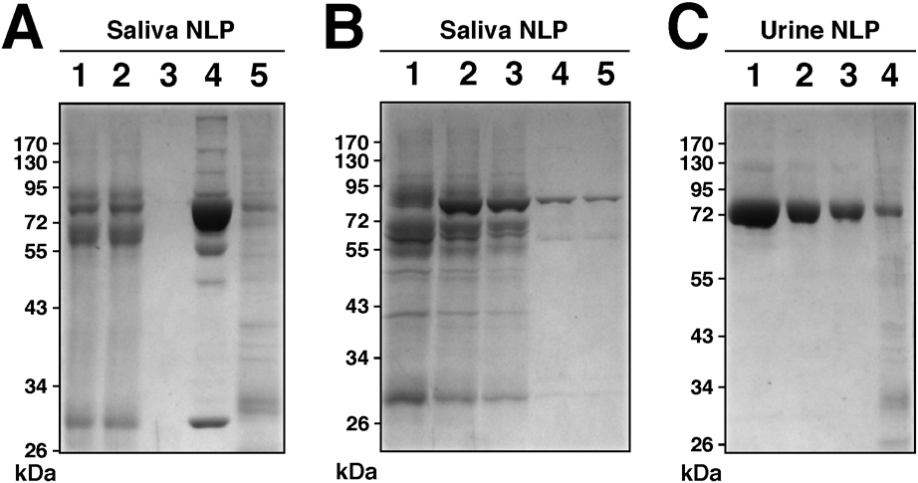
Protein profiles of saliva and urine NLP are determined by their passage through medium containing various amounts of serum. (A) Saliva NLP, obtained as in the *Materials and Methods*, were inoculated into serum-free DMEM for 1 day (lane 1), 2 days (lane 2), and 14 days (lane 3). NLP were then pelleted, washed, and introduced into DMEM containing 5% HS, and incubated for 1 day (lane 4) and 12 days (lane 5). (B) Saliva NLP were inoculated into DMEM containing 5% HS for 4 h (lane 1), then pelleted, washed, and inoculated into serum-free DMEM for 1 day (lane 2), 2 days (lane 3), 5 days (lane 4), and 6 days (lane 5). (C) Urine NLP inoculated into serum-free DMEM and incubated for 1 day (lane 1), 2 days (lane 2) and 4 days (lane 3), after which NLP were collected, washed, and reinoculated into DMEM containing 5% HS and incubated for 2 days (lane 4). Formation of NLP from saliva and urine, their processing and subculturing, and standardization of the protein contents loaded onto each lane are described in the *Materials and Methods.* Note the gradual fading of protein bands in serum-free medium as well as the acquisition of multiple protein bands in the presence of serum.

To further verify this point, namely that the protein profile of NB from any one of these fluids, not just serum, can be morphed according to the protein constituents in the surrounding milieu, the following serial passage experiments were done (Fig. 16). In Figure 16A, saliva NLP were inoculated into DMEM without serum for 1 day (lane 1), 2 days (lane 2), and 14 days (lane 3), which showed a gradual fading of the protein bands, probably due to the formation and enrichment of protein-free mineral complexes. The same saliva NLP maintained in serum-free medium were then washed and introduced into DMEM containing 5% HS, and gel profiles were again obtained after incubation for 1 day (lane 4) and 12 days (lane 5). Note that upon inoculation into serum, the protein-free complexes immediately acquired the same three major bands described before, along with some other minor bands, making these saliva NLP virtually indistinguishable from the serum-derived NLP or NB studied earlier. With continued incubation (12 days, lane 5), the protein profile became smeared, with the fading of the main 66–75 kDa and the acquisition of multiple other bands, similar to what we had observed earlier with serum NLP and NB.

Likewise, when saliva NLP were inoculated from the outset into DMEM containing 5% HS, a complex profile with multiple bands was seen (Fig. 16B, lane 1). These same NLP were then collected and inoculated into DMEM without serum, and over the course of the new 1–6 days, there was again a gradual fading of the bands until only the 66–75 kDa remained, along with the fainter 52–65 kDa and 27–33 kDa bands (Fig. 16B, with lanes 2–5 corresponding respectively to 1, 2, 5, and 6 days of incubation in serum-free DMEM).

This protein modulation by culture medium was not limited to saliva NLP and could be demonstrated with any other body fluid NLP. Figure 16C shows a representative experiment done with urine NLP inoculated into DMEM without serum and allowed to incubate for 1–4 days, whereby there was a gradual fading of the main 66–75 kDa as before (lanes 1–3 correspond to incubation for 1, 2, and 4 days, respectively). After 4 days of incubation, when the same NLP were collected, washed, and reinoculated into DMEM containing 5% HS, there was again binding to the same more prominent serum bands which became smeared with prolonged incubation (Fig. 16C, lane 4 corresponds to 2 days in DMEM with 5% HS). Taken together, the results presented here demonstrate that irrespective of the origin of any NB, be them from serum or from any other body fluid, their protein composition will largely mirror the protein constitution of the culture medium used for their maintenance and growth.

The exhaustive protein identification done here with NLP and NB derived from serum (a total of 142 trials) as well as other body fluids (a total of 57 trials) can be used to assert the conclusion that, in our hands, every single NB-associated protein found to date corresponds to eukaryotic proteins that have previously been characterized and identified in the respective compartmental fluids from which they were derived. This finding makes unlikely the possibility that any foreign proteins, including non-eukaryotic proteins, may be present in NB that cannot be explained by simple, physical binding by known proteins to calcium or apatite. This conclusion stands in marked contrast with the earlier inferences of novel prokaryotic proteins including porins and complex peptidoglycans being associated with NB [2], [60] and with later reports that have claimed to have identified specific NB-associated bacterial sequences like elongation factor Tu and GroEL [17].

### Immunological specificity of NB antibodies and cross-reactivity with albumin and fetuin-A

Monoclonal antibodies deemed specific for human NB, available through Nanobac Oy, are now routinely used in immunoblotting and tissue staining [2], [3], [16], [17], [34]. These same reagents have been used in numerous studies as confirmatory tools to establish the presence of NB in human diseased tissues. Table 2 provides a listing of some of these studies along with a description of the source of material used to generate NB such as the presence or absence of serum used for their culture, and the type of antibody as well as the immunological method employed to detect them. Given the critical importance of these reagents in the context of demonstrating the presence of NB in various human pathologies, it is surprising how little, if any, information is known about these monoclonal antibodies with regards to their immunological specificity. Most of these studies relied on techniques such as tissue immunostaining, ELISA, and dot blotting, which do not give information on the molecular weight or the specificity of the target antigen. Only a few studies have tried to address the specificity issue of the NB antibodies. For instance, Vali et al. [32] had shown that NB monoclonal antibodies 8/0 and 5/2 bind to two protein bands of 35 kDa and 66 kDa, with the latter attributed to albumin, which the authors discarded as non-specific binding. Miller et al. [16] showed reactivity of antibody 8D10 against one single band of 50 kDa from cultures of human calcified arteries deemed to contain NB. We, on the other hand, demonstrated that this antibody 8D10 as well as the other commonly used NB-specific monoclonal antibody 5/3 strongly react, in fact, with both human and bovine albumin [20]. In contrast, Raoult et al. [19] demonstrated that this same monoclonal antibody 8D10 reacted against fetuin-A present in “nanons.” Furthermore, polyclonal antibodies raised against fetuin-A also reacted against “nanons” maintained in the presence of 10% FBS as well as against human kidney stones [19]. These last two studies call in question the specificity of NB antibodies as well as the relevance and significance of previous immunostaining studies done with these same reagents. That monoclonal antibodies marketed as being specific for NB should react against common serum proteins like albumin and fetuin-A is disconcerting since one would have expected to find either bacterial or novel proteins identified with such “specific” reagents should the protein coating of NB be derived in fact from active bacterial protein synthesis, as initially inferred from the NB literature [2], [60]. Instead, these results lend support to the notion extended here that the protein coating of NB is derived from the simple absorption of serum or other compartmentalized fluid proteins to nucleating mineral complexes.

**Table 2:** NB literature review with focus on human tissue localization, pathology implication, and methods of culture and detection used for identification of NB.

Source of NB	Disease Implications	Culture Medium^a^			Immuno-detection		Ref.
		No Serum	Medium+ FBS	Medium+ γ-FBS	Method^b^	Antibody^c^	
Kidney stones	Kidney stone formation	+	−	+	IF	8/0, 5/2	[5]
Kidney stones	Kidney stone formation	+	−	−	None	None	[67]
Kidney stones	Kidney stone formation	+	+	−	WB, OI	Polyclonal	[15]
Kidney stones	Kidney stone formation	+	−	−	IF	8D10	[17]
Kidney tissues from cancer patients	Kidney stone formation	+	+	−	ELISA, IH	8D10	[34]
Kidney fluids from polycystic kidney patients	Polycystic kidney diseases	+	−	+	DB, IF	8/0, 5/2	[60]
Urinary tract stone from one astronaut	Urinary tract stone formation	+	−	+	IF	8D10	[71]
Urinary tract stones	None	−	+	−	IF	NM	[14]
Urine from patients with prostatitis	Prostatitis	−	−	+	IF	8D10	[72]
Calcified heart tissues	Pathological calcification	−	−	+	IF, IH	8D10	[16]
Calcified heart tissues	Pathological calcification	−	−	+	None	None	[37]
Calcified aortic heart valves	Heart valve calcification	−	−	+	None	None	[36]
Bile and serum from cholecystolithiasis patients	Cholecystolithiasis	+	−	+	IF, ELISA	NM	[73]
Bile from cholecystolithiasis patients	Gallstone formation	−	−	+	I-EM, IH	8D10	[18]
Nasopharyngeal carcinoma cell line	Nasopharyngeal cancer	−	−	+	IF	5/2	[74]
Joint fluids from arthritic patients	Arthritis	+	−	−	None	None	[75]
Sera from normal and diseased animals	None	+	−	−	None	None	[13]
FBS, human saliva, and dental plaque	None	+	−	+	None	None	[12]
FBS and human serum	Pathological calcification	+	−	+	IF	8/0, 5/2	[2]
FBS and cell lines	Cell culture contamination	−	+	−	None	None	[76]
FBS	None	−	+	−	I-EM, WB	8/0, 5/2	[32
NB strains DSM 5819-21	Pathological calcification	−	+	−	IH	8D10	[77]
NB strain Seralab 901045	None	+	+	−	WB, IF	8D10, polyclonal	[19]
NB strain Seralab 901045	Pathological calcification	−	+	+	IF	8/0, 5/2	[69]
NB strain Seralab 901045 and others	Pathological calcification	+	+	+	IF, WB	8/0, 5/2	[70]
NB strain Seralab 901045 and tumor tissues	Intratumoral calcification	+	−	−	IF	A4002	[78]

a DMEM was used in most studies; RPMI-1640 was used in the remaining few.

b Immuno-detection: immuno-fluorescence (IF), Western blotting (WB), Ouchterlony immuno-diffusion assay (OI), enzyme-linked immuno-sorbent assay (ELISA), immuno-histochemistry (IH), dot-blotting (DB), and immuno-gold TEM (I-EM).

c 8D10, 8/0, 5/2, and A4002 are commercial monoclonal antibodies raised against bovine NB (Nanobac Oy). Polyclonal antibodies were also generated against different NB cultures. NM: Not mentioned.

Since we [20] and Raoult et al. [19] obtained antibody reactivity against two different proteins (albumin in our study; fetuin-A in Raoult et al.'s) using the same NB-specific monoclonal antibody 8D10, we sought to reconcile this apparent discrepancy by studying the specificities of the various NB antibodies known to date. As albumin and fetuin-A are among the two main species that we have found associated with NLP/NB, we next sought to determine the immunological cross-reactivity between these two proteins in an attempt to explain why antibodies raised against NB cultured in FBS should be able to detect epitopes in human tissue antigens. Moreover, this analysis was further justified by the many studies published to date relying on these same antibody reagents to produce identification of NB in human diseased tissues. The rampant use of these reagents for such determination is problematic, to say the least, since it is clear that precipitating minerals bind to serum proteins present in the culture medium, casting doubt as what epitopes exactly, not to mention their species origin, the antibodies are really binding to.

**Figure 17:**
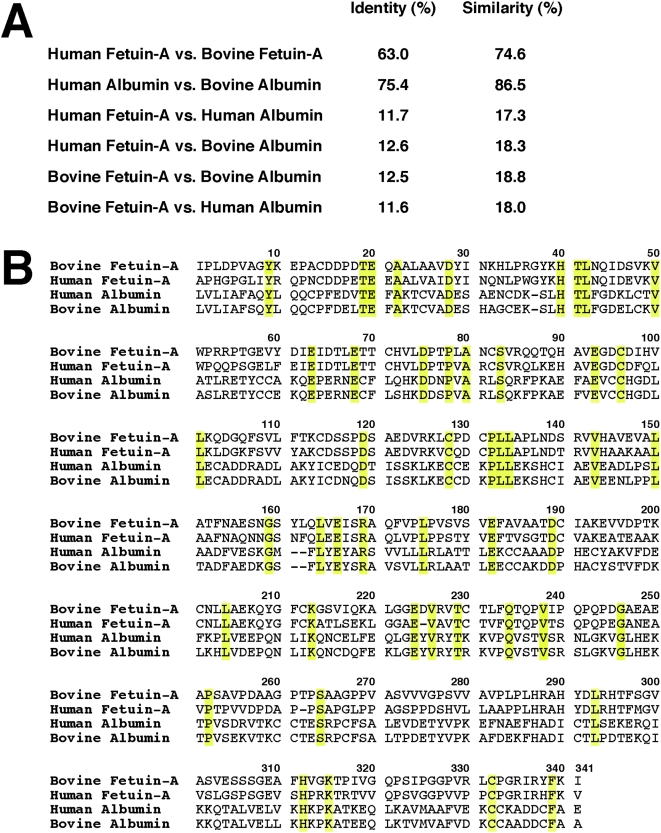
Sequence homology between fetuin-A and albumin. (A) Sequences were compared with the ClustalW program. The term “identity” refers to the presence of the same amino acids while “similarity” refers to the presence of different amino acids with similar chemical properties as assessed with the Gonnet matrix. Note limited homologies between fetuin-A and albumin from both bovine and human species. (B) Sequence alignment was done using the complete primary sequence of BSF (341 a.a.) as basis for comparison, while portions of the longer HSF (349 a.a.), BSA (583 a.a.), and HSA (585 a.a.) sequences were deleted at various sites for purposes of fitting the alignment. Identical amino acids are highlighted in yellow. The meter on top of the sequences refers to BSF only.

In line with this reasoning, it should be noted that fetuin-A (349 amino acids in the human form; 341 a.a., bovine) and albumin (585 a.a., human; 583 a.a., bovine) are known to share limited homology ranging from 11 to 13% identity and 17–19% similarity (Fig. 17A). The identity regions, while scattered throughout the length of the two sequences, appear to be a bit more clustered toward the N-terminus of both proteins (Fig. 17B). Accordingly, only fetuin-A is known to harbor a unique N-terminal cystatin-like domain which has been shown to be required for its high calcification inhibitory activity [25]. Mutational analysis and computer modeling of domain structures have suggested that a dense array of acidic residues on an extended β-sheet of the cystatin-like domain of fetuin-A mediates efficient inhibition of this type of mineralization [25]. The low homology between the two proteins may also explain earlier immunoblotting experiments that demonstrated weak immunological cross-reactivity between the two species that became apparent only following protein reduction and alkylation, which may have unfolded hidden homologies [61].

**Figure 18:**
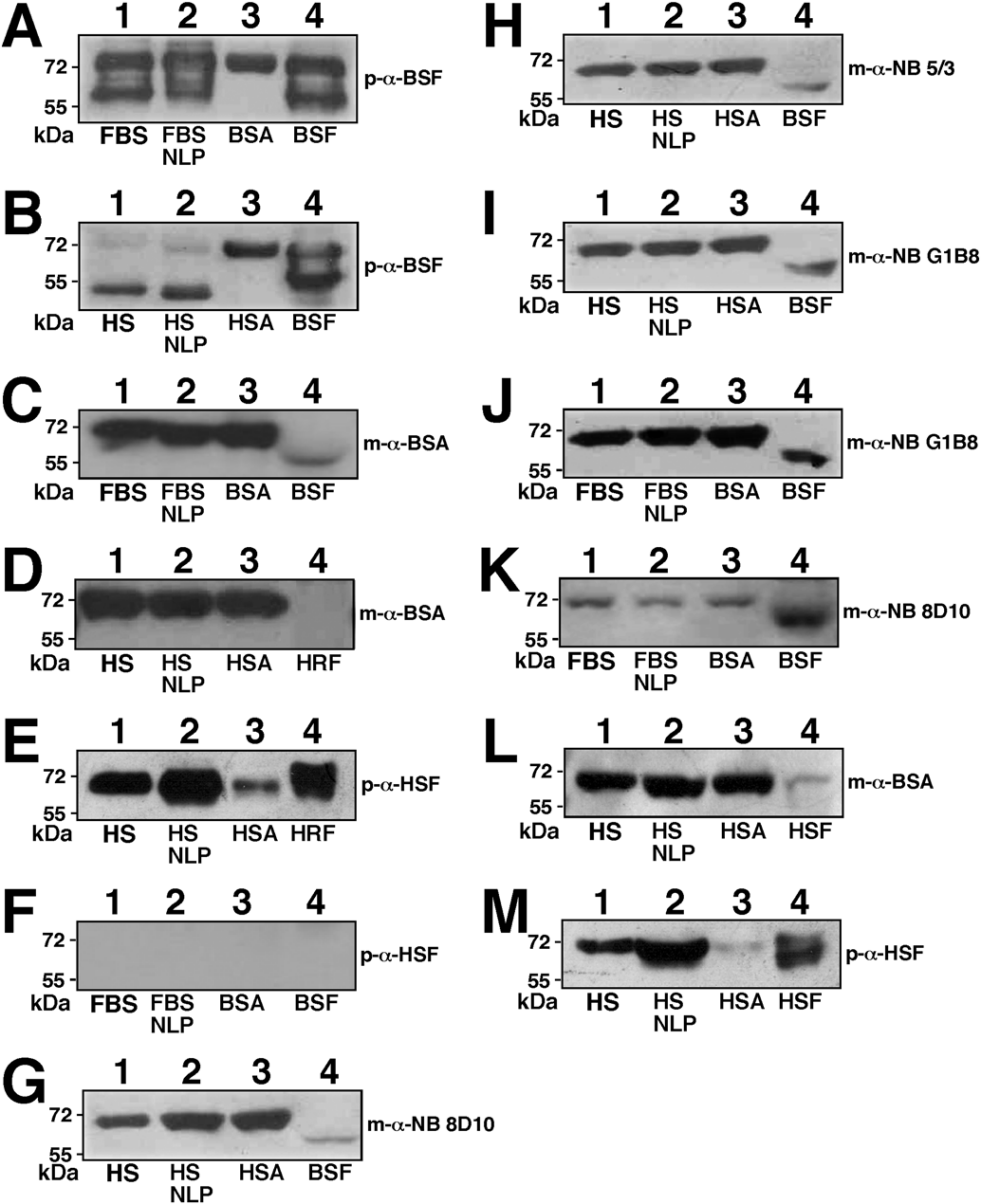
NB-specific antibodies cross-react with albumin and fetuin-A. Western blots on various bovine and human protein samples (bottom of each blot) were done using the indicated antibodies (to the right of each blot). Antigens used include FBS NLP and HS NLP obtained as in Fig. 14; purified BSA, BSF, HSF, and human recombinant fetuin-A (HRF); and whole FBS and HS. Antibodies used include a polyclonal antibody directed against BSF (p-α-BSF); a monoclonal antibody directed against BSA (m-α-BSA); a polyclonal antibody directed against HSF (p-α-HSF); and the monoclonal antibodies 8D10, NB 5/3, and NB G1B8 directed against bovine NB antigens (m-α-NB 8D10, m-α-NB 5/3, and m-α-NB G1B8, respectively). All gels were run under denaturing and reducing conditions except for (L and M), which were electrophoresed under non-reducing conditions.

We performed immunoblotting studies using three monoclonal antibodies claimed to be specific for NB as well as several polyclonal antibodies specific for serum albumin and fetuin-A (Fig. 18; see also *Materials and Methods*). As antigens, we used NLP obtained from both FBS and HS as well as commercially available purified albumin and fetuin-A (both human and bovine forms; in the case of fetuin-A, both purified protein and a human recombinant form were used). As positive controls, whole sera (HS and FBS) were also used. Given the limited immunological cross-reactivity known to exist between these two proteins [61], all the antigens tested were electrophoresed under reducing conditions using excess dithiothreitol (DTT) at 100 mM or 2.5% β-mercaptoethanol. As shown in Figure 18A, a polyclonal antibody raised against BSF, referred in the Figure as p-α-BSF, was shown to react with both FBS and FBS-derived NLP giving two prominent bands of 66–75 kDa and 52–65 kDa, when compared against the molecular weight standards used. This same antibody also reacted with bovine serum albumin (BSA) as a strong 66–75 kDa (Fig. 18A, lane 3). Against purified BSF used as control (Fig. 18A, lane 4), this polyclonal antibody reacted with at least two bands of 66–75 kDa and 52–65 kDa, similar to what was seen earlier with both FBS and FBS NLP (compare with lanes 1 and 2). In this respect, fetuin-A had earlier been shown to be heavily glycosylated, and this protein is known to assume multiple sizes when studied by SDS-PAGE [62]. The strong reactivity of this anti-BSF antibody against purified BSA (lane 3) suggests that bovine albumin and fetuin-A are antigenically cross-reactive under reducing conditions. However, the multiplicity of forms assumed by fetuin-A precludes us from drawing a firm conclusion as to whether the upper band seen with FBS and FBS NLP (lanes 1 and 2) represents cross-reactive albumin or a larger form of fetuin-A.

That such polyclonal antibodies prepared against BSF could cross species (bovine vs. human) was confirmed by using HS and HS-derived NLP as antigens (Fig. 18B, lanes 1 and 2). This time, weakly reactive bands were seen at the 66–75 kDa position, while a 52–65 kDa band reacted much more strongly. Controls using HSA as antigen again showed strong reactivity by a single band of 66–75 kDa (Fig. 18B, lane 3). It is clear from these immunoblots that the lower 52–65 kDa band can be assigned to fetuin-A (compare also with lane 4 containing BSF), whereas the upper 66–75 kDa band represents either albumin or fetuin-A in both the bovine and human species. However, it appears safe to conclude that the polyclonal antiserum prepared against bovine fetuin-A reacts with purified BSA, HSA and BSF, as well as with HSF found in both HS and NLP derived from HS. Together, these experiments reveal at least four levels of reactivity by a single antiserum, not only seen between different proteins of the same species but also with different proteins across species, illustrating the complexity of the NB phenomenology observed here as well as providing a basis for the ambiguous and problematic nature of the entire body of immunostaining data published to date on NB.

The type of cross-reactivity seen here could be extended to other antibodies, including monoclonals. Using a monoclonal antibody derived against BSA, referred as m-α-BSA in Figure 18C, we were able to react with the same bovine antigens used before in Figure 18A. Thus, FBS, FBS NLP, and BSA, used as antigens, all gave a single broad band migrating as 66–75 kDa (Fig. 18C, lanes 1–3). Against BSF, however, this monoclonal antibody reacted with a lower band of 52–65 kDa (Fig. 18C, lane 4). The same larger form of BSF seen earlier in Figure 18A was not found to react with this antibody, perhaps pointing to differences in immunological reactivity between the two antibodies. It is not clear also why this same antibody did not react with the 52–65 kDa form of BSF presumably present in FBS NLP (Fig. 18C, lane 2), as would have been expected from the reactivity seen with purified BSF (lane 4). When this same monoclonal m-α-BSA was used against human antigens similar to those used in Figure 18B, again single bands were seen (Fig. 18D, lanes 1–3) that were virtually identical to those seen with bovine antigens (compare lanes 1–3, Fig. 18D with Fig. 18C). However this antibody did not react against a human recombinant form of fetuin-A (HRF; see Fig. 18D, lane 4), suggesting that the protein species reactive in lanes 1–3 corresponds indeed to human albumin. Thus, using this monoclonal antibody specific for BSA, one can conclude that albumin is present in both bovine and human NLP. These results further illustrate the individual differences seen with each antibody used, substantiating our concern for unwarranted conclusions that may be drawn with any one of them in the absence of proper controls.

A third polyclonal anti-human fetuin-A, labeled as p-α-HSF in Figure 18E and F, produced yet another level of immunological cross-reactivity by binding to a single band of HS (Fig. 18E, lane 1), HS NLP (lane 2), and purified HSA (lane 3) as well as HRF which produced a much broader band, perhaps with multiple sub-bands (lanes 4). The same antiserum was also used against bovine antigens (Fig. 18F). It did not react with either FBS (Fig. 18F, lane 1), FBS NLP (lane 2), BSA (lane 3) or BSF (lane 4). These results reveal yet another pattern of antigenic reactivity not seen with the other antibodies.

Using three commercially available monoclonal NB antibodies (8D10, 5/3, and G1B8), we obtained similar reactivities against the same human antigens used earlier (Fig. 18G, H, and I, lanes 1–3). These antibodies reacted with HSA present as a 68–72 kDa band in the lanes corresponding to HS (lane 1 in all 3 Fig. 18G, H, and I), HS NLP (lane 2), and purified HSA (lane 3). Furthermore, all three monoclonal antibodies reacted with BSF (Fig. 18G, H, and I, lane 4), but not HRF (not shown). Virtually identical results were obtained with bovine antigenic material (Fig. 18J shows a blot reacted with monoclonal antibody G1B8 while Figure 18K shows reactivity with 8D10). Again, one single 66–75 kDa band was seen in FBS (lane 1 for both Fig. 18J and K), FBS NLP (lane 2), and BSA (lane 3), while BSF appeared as a lower band of 52–65 kDa (lane 4). We were intrigued that none of these monoclonal antibodies seemed to detect fetuin-A in FBS or FBS NLP but they seemed to react strongly to both BSA and HSA, suggesting that the monoclonals used here may have indeed been derived against albumin and that they may be only weakly cross-reactive against fetuin-A. Together, these results suggest that all three monoclonal antibodies previously deemed specific for NB react in fact with HSA, BSA and BSF, demonstrating again multiple levels of immunological cross-reactivity that may form the basis for understanding why these antibodies may have reacted against human antigens, results which in turn have been misinterpreted as confirmatory of the presence of NB in diseased tissues.

The immunological cross-reactivities seen here were not restricted to protein reducing conditions only, as otherwise expected from earlier published results [61]. In fact, similar antibody cross-reactivities were seen when proteins were electrophoresed under non-reducing conditions, as shown in Figure 18L and 18M for the monoclonal antibody specific for BSA (m-α-BSA) and the polyclonal antiserum specific for HSF (p-α-HSF). It is possible that the binding of these antigens to minerals may have resulted in conformational changes leading to the unfolding of primary sequences that in turn are reactive to antibodies even under non-reducing conditions.

The results seen here with NLP could be reproduced in their entirety with NB obtained from both FBS and HS as well as with the NB strains described earlier (data not shown). The results varied directly with the source of serum used for generating and maintaining NB. Accordingly, NB that had been propagated in serum-free medium showed progressively weaker antibody reactivities with prolonged incubation and serial passages before fading beyond detection (not shown).

Given the limited immunological cross-reactivity [61] and low levels of homology known to exist between albumin and fetuin-A, we did not expect the multiple cross-reactivities seen here with antigens probed under both reducing and non-reducing conditions. However, the data shown here should explain an entire body of spurious results exemplified by the many immunological detection studies published earlier, including the puzzling and seeming contradictory findings with NB-specific monoclonal antibodies shown to react with both HSA [20] and BSF [19]. Our results indicate that at least three distinct NB-specific monoclonal antibodies bind to epitopes shared by both albumin and fetuin-A. Since these two main serum proteins represent also the main constituents of NB, our results cast a new level of understanding with regards to what exactly previous immunostaining studies were detecting in human tissues.

Table 2 lists the various human tissues, along with their underlying pathologies, in which NB had been detected either based on morphological criteria alone or by using antibodies claimed to be specific for NB. For reference, the antibodies along with the various immunostaining techniques used are included in the same table. Note that all the NB-detection studies listed out in Table 2 have relied on culturing NB in FBS-containing medium and/or in medium alone. From the data presented here it should be obvious that, for some of the referenced studies in Table 2 that relied solely on morphological criteria to infer the presence of NB in human specimens as well as a role for NB in human diseases, the evidence presented is glaringly insufficient. Still from Table 2, it can be seen that γ-FBS has been used for culturing NB in an increasing number of recent studies relying on the assumption that this irradiated serum no longer provides a source of new NB while still serving as a nutrient source for NB growth [1], [2]. Given that γ-irradiation at the high levels (30 kGy or more) used by NB researchers has a drastic effect on proteins by inducing their aggregation, fragmentation and ionization [63], we would expect that such treatment would greatly reduce the biomineralization potential of FBS; thus, the results obtained with this system need to be viewed also with caution at this time.

As for the immunodetection studies listed out in Table 2, based on our own observations, it seems that, for NB cultures grown in the presence of FBS, the referenced studies had probably detected either BSA or BSF, or perhaps a combination of both. For serum-free cultures, the human counterparts of these same epitopes were presumably being detected; in such cases, it is also obvious that the initial seeding proteins would be diluted with each passage, until they would fade below detectable levels, as demonstrated by our own serial passage experiments. It is indeed problematic that antibodies deemed specific for NB should actually be detecting serum proteins not only of the same species but also across species. That is, several antibodies, including the ones used in our studies, were presumably derived against bovine NB but were also marketed as specific for human NB antigens, as claimed on their product specifications (Nanobac Oy). Besides the question of antigenic specificity, it is troublesome that antibodies reacting against protein epitopes present in the FBS (bovine origin) are being used to affirm the presence of human epitopes in diseased tissues, an unprecedented scenario that would question the validity of all the immunostaining studies published to date.

As an example, Khullar et al. [15], listed in Table 2, used polyclonal antiserum derived against NB originating from human kidney stones but that had been “grown” in RPMI 1640 supplemented with 10% FBS. It is thus impossible to ascertain the nature as well as the species of the epitopes being detected in all such past NB preparations collected from human tissues but that had been maintained in culture medium containing FBS or γ-FBS since any NB detected under such conditions would likely contain bovine antigens.

### Role of fetuin-A and albumin as both inhibitors and seeders of NB and NLP

**Figure 19:**
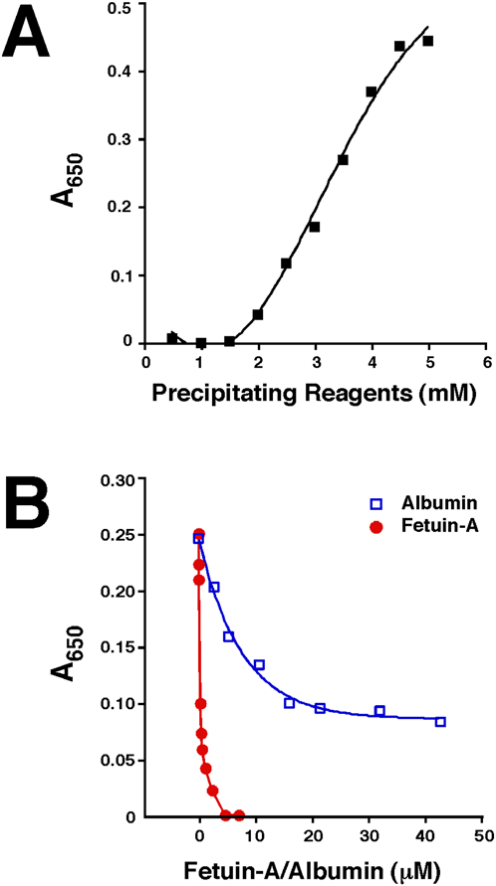
Inhibition of NLP formation by fetuin-A and albumin. (A) NLP were prepared from 0.5 mM to 6 mM each of CaCl_2_, Na_2_CO_3_, and NaH_2_PO_4_ added to water. A_650_ readings using a spectrophotometer show dose-dependent increase in optical density with NLP formation that displays a linear relationship seen between 2 and 4 mM of precipitating reagents used. (B) The inhibitory effects of fetuin-A and albumin on NLP formed in water were studied using 3 mM each of the three precipitating reagents. BSF or HSA were added to the indicated concentrations prior to the addition of the precipitating reagents. Compared to HSA, BSF showed markedly higher inhibitory potency on NLP formation.

Since both fetuin-A and albumin are known inhibitors of calcium biomineralization, we next sought to verify whether these same proteins can inhibit the formation of NLP. We first developed a simple turbidity assay based on the propensity of calcium, carbonate and phosphate to combine and form insoluble complexes that can be read at 650 nm by means of color spectrophotometry (*Materials and Methods*). Initial studies were met with variable results and wide margins of error which were corrected after the incubation times were properly controlled and the readings consistently done. As shown in Figure 19A, the concentrations of the various precipitating reactants (calcium, carbonate, and phosphate) could be conveniently adjusted and chosen to provide a dose-dependent reaction yielding a linear component stretching between 2 and 4 mM of calcium-carbonate-phosphate that could be used in turn to monitor the role of calcium-binding proteins. Based on these considerations, a final concentration of 3 mM was chosen for calcium, carbonate, and phosphate in the experiment shown in Figure 19B, while the amount of BSF or HSA was varied. The inhibition achieved with BSF was much more pronounced and efficient than that seen with HSA. The protein concentration needed to obtain 50% inhibition was estimated at 0.3 µM for BSF, compared to 6 µM for HSA. We found that Heiss et al. had earlier performed a comparable assay to study the formation of “calciprotein particles” that were obtained by mixing CaCl_2_ and Na_2_HPO_4_ in the presence of 50 mM Tris-HCl and 140 mM NaCl at pH 7.4 [58]. In their study, the concentration of bovine fetuin-A needed to obtain 50% inhibition was estimated at 7.4 µM, which is significantly higher than what we obtained here. This difference may be attributed in part to the presence of carbonate ions in our reaction assay which, through their own binding to calcium, may have enhanced the inhibition seen with the added proteins. Since carbonate groups appear to constitute an important part of the NB scaffold, they were included in our assay so as to reproduce more precisely the NB phenomenology.

**Figure 20:**
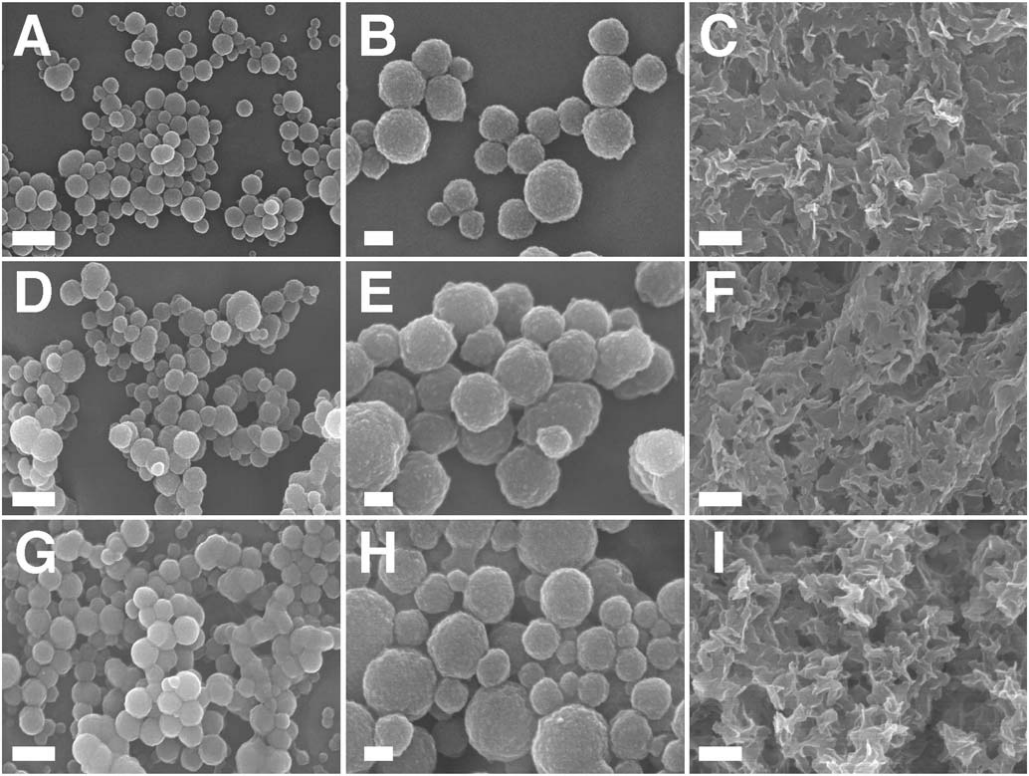
NLP associated with fetuin-A or albumin are morphologically similar. NLP were produced as described in Fig. 19, with 3 mM each of CaCl_2_, Na_2_CO_3_, and NaH_2_PO_4_ incubated with either 10 µM of BSF (A–C), 50 µM of HSA (D–F), or a combination of 10 µM of BSF and 50 µM of HSA (G–I). Incubation was done for 3 days (A, B, D, E, G, and H) or 1 week (C, E, and I) at room temperature. The initially clear solutions increased in turbidity with time, and by 3 days showed noticeable precipitation, which were then pelleted by centrifugation and washed with HEPES buffer once, followed by another wash with water, prior to SEM analysis. (B, E, and H) represent enlarged views of (A, D, and G), respectively. By one week of incubation, the round particles had coalesced to form films. Round particles or films seen with the 3 types of treatments appear virtually identical. Scale bars: 100 nm (B,D,F); 500 nm (A,C,E).

**Figure 21:**
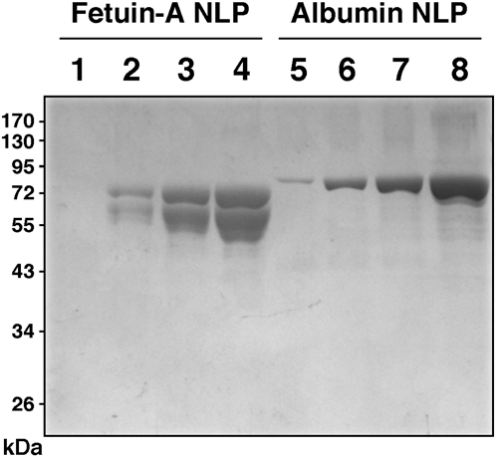
Demonstration by SDS-PAGE of the presence of fetuin-A and albumin in NLP formed from inhibitory complexes. NLP were prepared as in Figs. 19 and 20, from 3 mM each of CaCl_2_, Na_2_CO_3_, and NaH_2_PO_4_ in 1 ml of water incubated with either BSF or HSA at the following concentrations: 40 µg/ml, 80 µg/ml, 160 µg/ml, and 320 µg/ml of BSF, corresponding to lanes 1–4, respectively; and 0.2 mg/ml, 0.4 mg/ml, 0.8 mg/ml, and 1.6 mg/ml of HSA, for lanes 5–8, respectively. After an incubation of 3 days at room temperature, centrifuged pellets were washed three times with HEPES buffer, and the final washed pellets were resuspended in 50 µl of double-distilled water containing 50 mM EDTA, of which 16 µl were loaded onto each lane. Note the presence of multiple bands around 50–70 kDA associated with BSF and a prominent band centered around 72 kDa corresponding to HSA.

When the same incubation mixtures were allowed to sit at room temperature or at 4°C, we noticed precipitation that increased with time. In the case of albumin, precipitation could be noticed within 2 hours of incubation, which increased over the next 24 hours. With fetuin-A, precipitation was slower, becoming more pronounced after overnight incubation and which continued to increase for the next three days. When examined by SEM, both fetuin-A and albumin complexes showed typical round, amorphous NLP structures (Fig. 20A, B, D, E, G, and H) that further coalesced into film-like crystalline structures with incubation (Fig. 20C, F, and I). Precipitates obtained from the three different types of treatment produced virtually identical morphologies. That both fetuin-A and albumin were associated with these precipitates could be ascertained by SDS-PAGE, which demonstrated a dose-dependent increase in the amount of proteins found in the precipitating NLP as a function of the input protein used (Fig. 21).

These experiments suggest that fetuin-A and albumin can behave both as inhibitors of NLP formation as well as potential seeders. The initial inhibition demonstrated by the A_650_ turbidity measurements appears to have been overcome after a period of incubation, and this lag time is shorter with albumin as compared with fetuin-A. These observations are in line with earlier studies with fetuin-A which demonstrated that this potent serum calcium phosphate inhibitor may also function as a nidus of calcium complex formation and mineralization when the same inhibition is overcome [58], which would in turn explain the unusually high concentration of fetuin-A found in the bone matrix [64], while its absence in fetuin-A knocked-out mice is associated with ectopic calcification [65], two seemingly paradoxical findings that can be easily reconciled when the inhibition and seeding of calcium mineralization are seen as two sides of the same coin. That is, the same high affinities for calcium phosphate seen with fetuin-A, and albumin to a lesser extent, enable them to act as both inhibitors as well as nuclei for calcium binding and crystallization.

### Role of lipids in NLP and NB formation

In experiments resulting in the formation of NLP from serum, a milky type of coloration could often be seen associated with the NLP pellets and this material appeared to be of lipid origin. This was particularly noticeable with NLP derived from HS but was seen to vary with the serum lots used. The presence of lipid in NLP and NB was further suggested by the finding that apolipoprotein A1 is present in all the NLP and NB studied to date. Apolipoprotein A1 happens to be the primary protein constituent of high-density lipoproteins (HDL) [66], helping define the many physicochemical properties of HDL in the serum. Accordingly, lipids could be present either indirectly through the binding of apolipoprotein A1 to calcium phosphate complexes or directly through the binding of lipids to minerals, as proposed earlier by Cisar et al. [12], who demonstrated NB formation by inoculating pure phosphatidylinositol into DMEM without serum.

Preliminary lipid analysis using an enzymatic-colorimetric quantification method (*Materials and Methods*) revealed that NLP prepared from 1 ml of HS, diluted to 5% in DMEM with 5 mM each of calcium, carbonate, and phosphate, gave a reading of 3 µg of triglycerides and 1 µg of low-density lipoproteins (LDL). No HDL or cholesterol could be detected. Similar assays done on NLP produced from 1 ml of FBS revealed 2 µg of triglycerides but no measurable LDL, HDL or cholesterol. The amount of triglycerides and LDL detected for the NLP here represents a small fraction of the lipids present in normal serum. If we take the upper limits of 1.5 mg of triglycerides and 1.0 mg of LDL per ml of HS as references, the data shown here for HS NLP would indicate that less than 0.2% of the total triglycerides and less than 0.1% of the LDL in HS were precipitated through NLP. Alternatively, by means of an agarose gel electrophoresis kit used routinely in clinical laboratories for determining the profiles of more common serum lipoproteins like HDL and LDL (*Materials and Methods*), we could not obtain any detectable profiles. In contrast, untreated serum samples used as controls gave clear profiles that varied with the serum lots tested (not shown). It is not clear whether the low amounts of triglycerides and LDL detected here or the negative results seen with HDL may simply represent our own methodological limitations at this time since at least HDL would have been expected to be present given the presence of apolipoprotein A1 in all the NLP preparations studied to date. Still in this regard, it should be noted that our samples of human, but not bovine, NLP revealed consistently the presence of apolipoprotein B100, known structural component of LDL, which may explain the presence of detectable amounts of LDL and triglycerides in some of the assays performed for the human NLP. More definitive experiments are clearly needed to elucidate the lipid composition of these nanoparticles. The NB-seeding capability of the various lipid fractions found in normal serum must also be further determined. Given the structural basis already advanced here for the NB scaffold in terms of its protein and mineral composition, additional determinations can now be extended to encompass not only lipids but also nucleic acids, which we believe may also bind to NB leading in turn to the wide repertoire of ambiguous results reported before [1], [2].

### Conclusion

Together, these results confirm our notion that the presumed NB nidi or seeds contained in the serum may in fact be simple calcium or apatite-binding factors. These factors tend to sequester calcium and/or calcium phosphate, preventing precipitation and, with it, unnecessary calcification. That is, these inhibitors bind to the crystal nucleus in order to prevent further crystallization, and by doing so, they modulate the formation of small, round nanoparticles earlier misconstrued to represent a new form of bacteria. As such, these inhibitors appear to be part of the normal homeostatic mechanisms deployed by the various body fluids to regulate calcium and calcium phosphate. In fact, inadvertent mineralization in humans is known to be carefully prevented by calcification inhibitor proteins such as fetuin-A, albumin, matrix-Gla protein, osteopontin, and osteoprotegerin, among others (reviewed in references 38 and 59). While all serum-related experiments have consistently shown the presence of the three major proteins albumin, fetuin-A, and apolipoprotein A1, it is clear from this study that these proteins need not be exclusively present in all forms of NB, as demonstrated by the NB specimens obtained from other body fluids, which in fact revealed the presence of different proteins. The divergence in the proteins found associated with the various NB scaffolds studied here confirms further the notion that the presence of such proteins and the consequent formation of NB are probably circumstantial, depending merely on their availability in the local milieu. In fact, factors other than proteins may also be involved in generating NB as demonstrated through our trypsin-serum experiments. In this sense also, we raise caution concerning the use of the term “nanons” or fetuin-mineralo complexes to describe NB, as advanced by Raoult et al. [19], since their use presumes the identification of a novel protein-related entity with pathogenic potential, which the authors suggested to be comparable to prions. In our own hands, not only is fetuin-A not always the major protein species when compared with albumin and apolipoprotein A1, but in our mind, these NB complexes represent simple bystander products produced through binding to calcium and apatite. In fact, results from the simple experiments shown here in our study can probably explain all the main findings reported in the NB literature, including the marked pleomorphism associated with NB.

NB have been described as potential pathogens with the ability to initiate extraskeletal calcification *in vivo*, and recent reports have flagged alarm concerning their widespread presence, filterability, self-replication, and blood-borne infectivity [see for example refs. 4], [7], [and 49]. Numerous other studies have suggested a role for NB as causes for calcification-related pathologies, as exemplified by the formation of Randall's plaque in renal papillae deemed to be involved in the subsequent formation of kidney stones [34] or atherosclerotic plaques in blood vessels [16]. As sources of transmissible pathogenicity, NB have been deemed to satisfy in part the Koch postulates [4], [18], [37], [67]. In our view, however, a role for NB in pathogenesis, if any, is devoid of any substantive meaning unless NB can be defined chemically and structurally first, which we have sought to define here in this study. As pointed out by Urbano and Urbano in their clearly articulated and critical evaluation of the NB literature [11], the same Koch postulates used earlier to substantiate the germ theory were predicated on well-defined biological cycles and structures advanced for the microbial pathogens in question, all of which are still lacking for NB. This uncertain scenario is compounded by the many immunostaining studies done in the past using antibodies that are now known to bind not only to antigenic epitopes of common serum proteins but that, even more seriously, may have inadvertently been used across species. That is, our study here shows that human NB were most likely being detected with antibodies raised against bovine albumin or fetuin-A, and likewise, NB presumably originating from human tissues will most likely acquire bovine antigens when the same tissue homogenates are transferred to FBS-containing medium. These considerations alone would probably render the entire body of NB literature using these reagents largely uninterpretable.

The results shown here suggest instead that the NB phenomenology is likely an epiphenomenon stemming from normal calcium homeostasis. That is, calcium-binding and inhibitory factors normally present in serum and other body fluids should bind to any excess calcium or apatite present in the medium, allowing the calcium-bounded complexes to be siphoned for clearance and body disposal. In this sense, the same inhibitory and seeding factors must be readily available in the serum as well as in the other body fluids. According to the mechanism proposed here, these same calcium-binding inhibitory factors, be them fetuin-A, albumin, or other binding factors, should sequester increasing amounts of calcium or apatite by binding to them, until saturation is reached, at which point the same inhibitory complexes would aggregate, become insoluble, and behave as seeds or nidi for further binding of apatite until microscopic NB are formed. In the case of *in vitro* cell culture devoid of a proper clearance mechanism, these by-products would accumulate, thereby giving rise to the entire NB phenomenology. In retrospect, the original discovers of NB may have rediscovered the phenomenon of mineral or protein-mineral self-aggregation, which is probably ubiquitous in nature, as we and others have seen with calcium and silicate compounds [20], [28]–[31].

The question remains whether this same phenomenology could in turn have any pathogenic implications for the body. In this respect, it would seem that any overt accumulation of unwanted debris could in principle generate deleterious effects on the body physiology, and the continuous accumulation of self-propagating protein-mineral complexes should not be viewed differently. Thus, the same inhibitory proteins deployed by the body to clear excess calcium or nascent apatite may also in principle serve as seeds or nidi for extraskeletal calcification, a notion supported by this study. At this juncture, we are impelled to turn to the insightful overview on calcium granulations written by Ryall [68], who emphasizes through multiple examples that such calcium-deposit structures can be seen across a multitude of lifeforms in nature. Using the vast body of knowledge accumulated on calcium granulations, the same author questions whether similar bodies seen in human tissues, like Randall's plaques, are really the precursors of pathologies or perhaps, as she infers, they may represent innocuous or byproduct calcium deposits that are part of a more general calcium homeostatic and clearance cycle. In line with this reasoning, we also prefer to look at NB as simple calcium granulations deployed by the body as part of a normal calcium clearance mechanism that may have been inadvertently amplified through static cell cultures or in some body conditions which ended up turning the same inhibitory calcium complexes into seeds for apatite precipitation.

## Materials and Methods

### Culture of NB

NB were isolated from human serum as described earlier [2]. Briefly, human blood was obtained from healthy human volunteers by a conventional venipuncture technique. The use of human samples in this study was approved by the Institutional Board of Chang Gung Memorial Hospital. Written informed consents were obtained from the individuals who provided the samples. Whole blood was withdrawn into Vacutainer tubes without anticoagulant (Becton, Dickinson & Company, Sparks, MD, USA). Blood samples were centrifuged at 1,500×*g* for 15 min at room temperature. The supernatant corresponding to the human serum was then successively filtered through 0.2 µm sterilizing filters, diluted 1∶10 to 1∶300 in DMEM (Gibco, Carlsbad, CA, USA), followed by incubation at 37°C in cell culture conditions for several weeks. The criteria used to confirm a positive culture of NB were based on earlier studies [1], [2]. These criteria included the formation of a visible white precipitate on the culture flask; increase of optical density of the culture medium and slow doubling time of 3 days as measured by A_650_; the presence of round particles with sizes ranging from 50 to 500 nm as seen under TEM and SEM; the detection of high peaks of calcium and phosphorus by EDX; and a positive reaction against commercial NB antibodies (Nanobac Oy, Kuopio, Finland).

NB were also prepared from various body fluids (saliva, urine, ascites, cerebrospinal fluid or CSF, pleural effusion, and synovial fluid), that had been filtered successively through 0.2 µm and 0.1 µm membranes, by means of their inoculation into serum-free DMEM at 1∶10 dilutions. Cultures were analyzed for the appearance of NB as above. Precipitates could be seen within 3–7 days of culture. Saliva was obtained from healthy volunteers. Urine samples were obtained either from healthy volunteers or from patients (4 samples) with nephrotic syndrome, provided by Dr. See-Tong Pang, Department of Urology, Chang Gung Memorial Hospital (CGMH), Kuei-Shan, Taiwan. The other body fluids (5 samples of each) were provided by Dr. Kuo-Chien Tsao, Department of Clinical Pathology, also of CGMH. These body fluids were obtained from patients with various clinical conditions.

Three strains of NB, designated as DSM 5819, DSM 5820, DSM 5821, were obtained from the German Collection of Microorganisms and Cell Cultures (DSMZ; Braunschweig, Germany). DSM 5819–21 were initially isolated from different lots of commercially available FBS [69]. These strains were later deposited by Olavi E. Kajander (Nanobac Oy) into the DSMZ in association with a patent issued in 1992 describing the isolation of NB (USA patent #5,135,851). Another strain of NB designated as “Nanobacterium sp. strain Seralab 901045” was provided by Dr. Didier Raoult (Unité des Rickettsies, Centre National de la Recherche Scientifique UMR 6020, Faculté de Médecine, Marseille, France). This strain was also initially isolated from FBS (Seralab, Sussex, UK ; lot. #901045 ; see reference 70); it was later referenced as “nanons” by Raoult et al. [19]. All strains of NB were cultured as described earlier [70], using DMEM with or without FBS (Biological Industries, Kibbutz Beit Haemek, Isreal ; PAA Laboratories, Pashing, Austria). For our experiments, subcultures were performed by diluting a one-month old culture in a ratio of 1∶10 in fresh DMEM with or without FBS. Cultures were maintained in a cell culture incubator at 37°C in humidified 5% CO_2_-95% air.

### Preparation and culture of NLP

All solutions used for the precipitation experiments were filtered through 0.1 µm filters prior to use. NLP were prepared by successively adding solutions of 0.25 M CaCl_2_, 0.25 M NaHCO_3_, and 0.25 M Na_2_HPO_4_, all adjusted to pH 7.4, to DMEM, to a final concentration of 0.1 to 10 mM each. In some experiments, either FBS or HS, that had been filtered successively through 0.2 µm and 0.1 µm membranes was inoculated into DMEM to a final concentration of 0.3–30% before the final addition of the 3 precipitating reagents. For NLP obtained from body fluids, except indicated otherwise, these fluids were treated directly with the 3 precipitating reagents to a final concentration of 8 mM each. For FBS or HS NLP used for SDS-PAGE, either 1% or 5% serum was used and the precipitating reagents were always added to 3 mM. Alternatively, NLP were prepared by adding 1 M CaCl_2_ and 1 M (NH_4_)_2_CO_3_ to DMEM to a final concentration of 10 mM. NLP formation under these conditions was not a simple result of pH changes since at 10 mM each of CaCl_2_ and (NH_4_)_2_CO_3_ in DMEM, the pH increased from 7.4 to 7.7. For the experiment shown in Fig. 2A, calcium carbonate particles were obtained by adding 50 mM each of CaCl_2_ and (NH_4_)_2_CO_3_ into DMEM, which slightly increased the pH from 7.4 to 7.8. The precipitation solutions were incubated with end-over-end shaking for 30 min at room temperature. NLP were then pelleted by centrifugation in a micro-centrifuge at 16,000×*g* for 10 min at room temperature. NLP were washed twice with either HEPES buffer (20 mM HEPES, 1 mM CaCl_2_, 2 mM Na_2_HPO_4_, 0.02% sodium azide, and 0.15 M NaCl, pH 7.4), DMEM, or double-distilled water, using the same centrifugation step. Pelleted NLP were finally used as such or re-suspended in either HEPES buffer, DMEM, 50 mM EDTA, or double-distilled water for SDS-PAGE and the different analyses described below.

NLP prepared in DMEM as described above were cultured at 37°C in cell culture conditions in 24 well plates. NLP were propagated by sub-culturing 2 day-old NLP 1∶10 into 1 ml of fresh DMEM without serum. In some experiments, specified amounts of HS or FBS were present in the DMEM used. The effect of trypsin on the formation of serum-derived NLP was studied by adding this enzyme (stock solution of 5% in DMEM, pH 7.4) to a final concentration of 0.5% in serum-containing DMEM, followed by incubation at 37°C for 2 hours prior to the addition of NLP precipitating reagents. The effect of EDTA and EGTA was studied by diluting them separately in DMEM at final concentrations of 10 mM prior to addition of the precipitating reagents. The effects of magnesium and carbonate on NLP formation were evaluated by adding MgCl_2_ or NaHCO_3_ from 0.25 M stock solutions at pH 7.4 to DMEM at final concentrations ranging from 1 to 10 mM for magnesium and 1 to 80 mM for carbonate, prior to the addition of calcium and phosphate. To evaluate the effect of temperature on NLP formation, some plates were also incubated at room temperature or 4°C, in normal atmospheric conditions.

### Photography and spectrophotometry

Pictures of 24 well plates were acquired from underneath using a scanner in reflective light mode (Scan Maker 8700, MicroTek, Hsinchu, Taiwan). The plates taken from the cell culture incubator were equilibrated at room temperature for 15 minutes prior to photography in order to remove condensation on the lid. Spectrophotometry readings of 24 well plates and 1 ml cuvettes were carried out using a Spectra Max M2 spectrophotometer (Molecular Devices, Sunnyvale, CA, USA) in absorbance mode at a wavelength of 650 nm. The plates were agitated inside the spectrophotometer for 10 seconds prior to each reading in order to ensure consistent readings. Data acquisition was performed with the SoftMax Pro 4.6 software (Molecular Devices). A_650_ readings averaging three different experiments were used to plot the A_650_ graphs.

### Energy-dispersive X-ray spectroscopy

Aliquots of washed NLP and NB re-suspended in water were deposited on 200 mesh formvar carbon-coated grids and dried overnight under a laminar flow hood. EDX spectra were acquired with a SEM S-3000N scanning electron microscope (Hitachi Science Systems, Tokyo, Japan) equipped with an EMAX Energy EX-400 EDX device (Horiba, Tokyo, Japan). Data acquisition was performed with the EMAX software (Horiba). Data acquisition time was fixed to 30 seconds in point mode analysis. At least three different areas were analyzed to ensure homogenous readings.

### Fourier-transformed infrared spectroscopy

NB cultured at 37°C in cell culture conditions for 2 weeks were scraped from the flasks, centrifuged using a L8-80M ultracentrifuge (Beckman, Fullerton, CA, USA) at 20,000×*g* for 1 hour. The samples were washed twice with double-distilled water using the same centrifugation step. NLP prepared as mentioned above were centrifuged at 16,000×*g* for 10 minutes and washed twice with double-distilled water. The washed pelleted particles were dried in a vacuum centrifuge for 15 minutes at room temperature. The dried powder was mixed 1∶100 (w/w) with KBr and compressed into a 1.3 cm diameter pellet using a hand press. FTIR spectra were obtained using a Nicolet 5700 FTIR spectrometer (Thermo Fisher Scientific, Waltham, MA, USA) equipped with a deuterated triglycine sulfate (DTGS) detector. Spectra were obtained at a resolution of 4 cm^−1^ at wavelengths spanning from 4000 to 400 cm^−1^, each time averaging 32 scans. The following commercial reagents were used for FTIR as well as the other analyses: calcium carbonate (A.C.S. grade reagent, purity 99.6%, Mallinckrodt Baker, Inc., Phillipsburg, NJ), calcium phosphate tribasic (Kanto Chemical Co., Tokyo, Japan) and HAP (buffered aqueous suspension, 25% solid, Sigma).

### Micro-Raman spectroscopy

Aliquots of washed nanoparticles were deposited on glass slides and dried overnight under a laminar flow hood. Micro-Raman spectroscopy analysis was carried out using the inVia Raman confocal microscope (Renishaw, Stonehouse, UK). The microscope was equipped with a 50× objective and a charge-coupled device (CCD) detector. The Raman excitation source consisted of a 633 nm laser beam operating at a beam power of 17 mW focused on the powder specimens.

### Scanning and transmission electron microscopies

For SEM, washed samples were re-suspended in double-distilled water and deposited on formvar carbon-coated grids. Excess liquid was removed with an absorbent paper and the grids were dried overnight under a laminar flow hood. The grids were coated with gold for 90 seconds. Specimens were observed with a field-emission SEM S-5000 scanning electron microscope (Hitachi Science Systems, Tokyo, Japan). For TEM, washed nanoparticles were also deposited on formvar carbon-coated grids. 1% phosphotungstic acid used as the negative-stain was applied for 90 seconds. Excess liquid was removed and the grids were dried overnight under a laminar flow hood. For thin-sections, the specimens were washed twice in double-distilled water and dehydrated with two washes of 100% ethanol. The samples were then embedded in Epon 812 resin (Electron Microscopy Sciences, Fort Washington, PA, USA) and incubated at 72°C for 2 days to allow resin polymerization. Thin-sections were cut using a Leica Ultracut UCT microtome and were transferred on formvar carbon grids. Negative-stain and thin-sections specimens were observed with a JEOL JEM-1230 transmission electron microscope (JEOL, Tokyo, Japan) operated at 120 keV.

### X-ray and electron diffraction analyses

Nanoparticles washed in water were deposited on glass slides and dried in a laminar flow hood overnight. XRD spectra were obtained using a D5005 X-ray diffractometer (Bruker AXS, Madison, WI, USA) with an X-ray copper tube operating at 40 kV. A secondary monochromator was used in combination with a scintillation counter detector. Data were collected over a 2θ angle ranging from 5 degrees to 80 degrees at a speed of 0.02 degree per second. Diffraction spectra were compared with the database of the Joint Committee on Powder Diffraction and Standards (JPCDS) to identify the chemical formula of the crystalline compound. X-ray diffraction patterns were acquired with a JEOL JEM-1230 transmission electron microscope operating at 120 keV.

### Sodium dodecyl sulfate polyacrylamide gel electrophoresis

Nanoparticles re-suspended in 50 mM EDTA were dissolved in 5× “loading buffer” (0.313 M Tris-HCl pH 6.8, 10% SDS, 0.05% bromophenol blue, 50% glycerol, 12.5% β-mercaptoethanol) to a final 1× loading buffer strength, heated at 95°C for 5 min, and resolved on a 10% SDS-PAGE using a mini-gel system (Hoefer, Holliston, MA, USA). In some experiments, reduction was achieved with 0.1 M DTT instead of β-mercaptoethanol. For Fig. 11, NB samples (2 ml) were prepared from a 1 month-old culture of DMEM containing 10% HS that were pelleted by centrifugation at 16,000×*g* for 10 minutes at room temperature, washed twice with HEPES buffer, as described earlier, and resuspended in 50 µl of 50 mM EDTA. “Nanons” and DSM 5819, having reached comparable turbidities as given by an A_650_ of about 0.6, were subcultured 1∶10 in serum-free DMEM or DMEM containing 10% FBS for 2 days, pelleted from a total of 2 ml, washed twice in HEPES buffer and processed as described for the other NB. NLP were prepared from DMEM containing either 1% or 5% serum (FBS or HS) inoculated with 3 mM each of calcium, carbonate and phosphate, incubated and processed as before. Either 8 µl of washed FBS NLP, HS NLP, HS NB, and DSM 5819, or 16 µl of “nanons” were loaded onto each gel lane. For NLP obtained from body fluids (Fig. 15), 600 µl of each fluid treated with 8 mM of each one of the 3 precipitating reagents, were pelleted as before, washed twice with HEPES buffer and resuspended in 50 µl of 50 mM EDTA, of which 14 µl were used for each gel lane.

For Figs. 13, HS NB, DSM 5820, DSM 5821, FBS NLP, and HS NLP reaching comparable densities (as judged by a turbidity of about 0.6 measured by A_650_ as well as by the size of pellets) were obtained from the indicated cultures and subcultured 1∶5 into the specified medium in 25 ml T-flasks. At the days indicated in the Figure legend, 1 ml of each culture was pelleted at 16,000×*g* for 10 minutes at room temperature, washed twice with HEPES buffer, and resuspended into 16 µl of 50 mM EDTA before applying onto gels. For Fig. 16, saliva NLP and urine NLP, obtained through 1∶10 dilutions in serum-free DMEM, were also collected when reaching an A_650_ of about 0.6 and subcultured 1∶5 into the indicated medium (serum-free medium or medium containing serum) for the specified periods of time, after which 1 ml of each culture was collected, pelleted, washed, and resuspended into 16 µl of 50 mM EDTA before applying onto gels, as before.

For the protein profile of FBS and HS depicted in Fig. 12 and the Western blot experiments shown in Fig. 18, the concentration of proteins loaded onto each lane was evaluated using the Bradford colorimetric protein assay (Biorad, Hercules, CA, USA). For Western blots, 0.5 µg of various proteins was applied to each lane. In the absence of antibody reactivity, the amount of proteins was increased to 5 µg (Fig. 18D, lane 4; and Fig. 18F). Gels were stained with Coomassie blue for 1 hour and destained in 40% methanol/7% acetic acid for 1 hour, followed by several changes of 5% methanol/7% acetic acid. The PageRuler prestained protein ladder (Fermentas, Burlington, Ontario, Canada) was used as molecular weight reference. Purified proteins used for some gels as well as for the Western blots included HSA (Sigma), BSA (Sigma), BSF (Sigma), HRF (R&D Systems, Minneapolis, MN, USA). Gels were photographed in black-and-white using the Multi Image Light Cabinet (Alpha Innotech Corporation, San Leandro, CA, USA).

### Matrix-assisted laser desorption ionization-time of flight (MALDI-TOF) mass spectrometry

Protein bands from Coomassie blue stained SDS-PAGE were excised with sterile truncated 200 µl pipette tips and destained twice in a 3∶2 mixture of 50 mM NH_4_HCO_3_:100% acetonitrile (ACN). The bands were dehydrated in 100% ACN and dried for 10 minutes in a vacuum centrifuge. The proteins were then reduced with 25 mM NH_4_HCO_3_ containing 10 mM DTT at 56°C for 45 minutes, and alkylated in 25 mM NH_4_HCO_3_ containing 55 mM iodoacetamide (IAA; Sigma) at room temperature for 30 minutes. The proteins were in-gel digested with sequencing grade modified porcine trypsin (20 µg/ml; Promega, Madison, WI, USA) overnight at 37°C. Peptides were extracted with 100% ACN containing 0.1% (v/v) formic acid and 0.5 µl of the peptides mixture was deposited on a MTP AnchorChip™ 600/384 TF (Bruker Daltonik, Bremen, Germany). Mass spectrometry (MS) and MS/MS spectra were obtained with an Ultraflex TOF-TOF mass spectrometer (Bruker Daltonics). Selected peaks were fragmented by the LIFT method to confirm the identity of the highest score protein by MS-MS analysis.

Alternatively, NLP samples were submitted to in-solution trypsin digestion. FBS NLP and HS NLP were prepared in the presence of either 0.3%, 1%, 3%, or 10% serum using 10 mM each of the three precipitating agents (CaCl_2_, NaHCO_3_, and Na_2_HPO_4_ in DMEM) as described earlier. NLP washed twice in DMEM and once in distilled water were successively reduced, alkylated, and digested with trypsin directly in solution, as described earlier. Peptides dried in a vacuum centrifuge were resuspended in 0.1% formic acid (Sigma), loaded onto a reversed-phase liquid chromatography trap column (Zorbax 300SB-C18, Agilent Technologies, Wilmington, DE, USA), and separated using a 10-cm analytical C_18_ column (New Objective, Woburn, MA, USA) by eluting with a succession of different linear gradients of increasing concentration of elution buffer (99.9% ACN containing 0.1% formic acid). The liquid chromatography column was coupled to a 2-D linear ion trap mass spectrometer (LTQ-Orbitrap; Thermo Fisher Scientific) operated using the Xcalibur 2.0 software (Thermo Fisher Scientific). Both MS and MS/MS spectra were acquired using one microscan with a maximum fill-time of 1000 and 100 ms for MS and MS/MS analysis, respectively.

### Mass fingerprint analysis

Peptide masses were annotated with the FlexAnalysis 2.2 software (Bruker Daltonics) with the following parameters: peak detection algorithm, SNAP; S/N threshold, 2; maximal number of peaks, 200. The resulting peak list was used for search against the non-redundant Swiss Prot database with the software BioTools 2.2 (Bruker Daltonics) and the MASCOT engine. The parameters used for the MASCOT search included carbamidomethyl cysteine and oxidized methionine as fixed and variable modifications, respectively. One missed cleavage was allowed. Peptide masses corresponding to human keratins were excluded. The parameters for MS/MS analysis were similar to the ones mentioned above, except that the S/N threshold was set to 5, the maximum number of peaks allowed was 100, and the fragment tolerance was 0.6 Daltons. The criteria for positive protein identification included a score above the MASCOT threshold of chance expectation value (p<0.05), a molecular weight corresponding to the band obtained on the SDS-PAGE, and a confirmation of the highest score protein by MS/MS analysis of two selected peaks.

For in-solution trypsin digestion, the Scaffold software (version 2.1.03, Proteome Software, Portland, OR, USA) was used to validate protein identification. Criteria for validation included identification of at least 3 peptides per protein and a probability of protein and peptide identification above 95%.

### Sequence alignment of fetuin-A and HSA

The protein sequences were retrieved from the NCBI protein database. HSF (NCBI accession number NP-001613), BSF (NP-776409), HSA (AAA98797), and BSA (AAA51411) protein sequences were compared and aligned using the ClustalW program with default parameters. The similarity between non-identical amino acids was evaluated using the Gonnet sequence similarity matrix of the ClustalW program. Due to the differences in the length of the proteins compared, we used the shorter BSF as the reference sequence against which the three other sequences were aligned and compared (Fig. 17B). This type of juxtaposition resulted in interruption of sequences in HSF, BSA and HSA that were manually removed for purposes of showing identical or homologous sequences only. Thus, for these three other sequences shown in Figure 17B, the sequence metering shown for BSF does not apply.

### Western blotting

Proteins resolved by SDS-PAGE were transferred to an Immobilon-P polyvinylidene fluoride (PVDF) membrane (Millipore, Bedford, MA, USA) at 400 mA at 4°C for 2 hours. The membrane was blocked in 5% defatted milk diluted in PBST buffer (PBS 1×, 0.1% Tween 20) for 1 hour. The antibodies used were sheep polyclonal anti-BSF (Novus Biologicals, Littleton, CO, USA), mouse monoclonal anti-BSA (Santa Cruz Biotechnology, Santa Cruz, CA, USA), goat polyclonal anti-HSF antibody (Santa Cruz Biotechnology), mouse monoclonal anti-NB antibodies 8D10, 5/3, and G1B8 (Nanobac Oy), and horse-radish peroxidase (HRP)-conjugated anti-mouse, anti-goat, and anti-sheep secondary antibodies (Santa Cruz Biotechnology). The three NB-specific monoclonal antibodies used were supposedly raised against bovine NB, but were also claimed to be specific for human antigens (Nanobac Oy). Primary antibodies were diluted 1∶1,000 in blocking solution and incubated with the membrane at 4°C overnight or at room temperature for 1 hour with orbital shaking. Membranes were washed for 5 minutes three times in PBST buffer. Secondary antibodies were diluted 1∶2,000 in blocking solution and incubated 1 hour at room temperature with shaking. The blots were revealed using the ECL western blotting detection reagents (Amersham Biosciences, Buckinghamshire, UK). The light signal was recorded by placing the membrane in contact with an autoradiographic film (Molecular Technologies, St-Louis, MO, USA) developed in a dark-room.

### Inhibition of NLP formation by fetuin-A and albumin

NLP were prepared by successively adding aliquots of individual solutions of 0.25 M CaCl_2_, 0.25 M Na_2_CO_3_, and 0.25 M NaH_2_PO_4_, all adjusted to pH 7.4, to water to final concentrations each ranging between 0 and 6 mM. The NLP solutions were prepared as before, and after transferring to transparent plastic cuvettes, the optical density of the solutions was monitored by reading the A_650_ with a spectrophotometer. To prevent data scatter, all readings were done within the shortest possible period of time, through standardization of the handling steps, including the resident time allowed for the solution to sit in the cuvette prior to spectrophotometric reading (less than 10 seconds). We noticed that this resident time, if not properly controlled, was a source of wide margin of error, most likely due to the inherent variability seen with particle suspensions.

To test the effect of fetuin-A and albumin on the formation of NLP, each one of the precipitating reagents (CaCl_2_, Na_2_CO_3_, and NaH_2_PO_4_) was added to a final concentration of 3 mM, within the linear range established for the assay. Stock solutions of BSF (purified from FBS; obtained from Sigma) or HSA (purified from HS; Sigma) were prepared in water at a final concentration of 20 mg/ml. BSF and HSA were added to water, followed by vigorous shaking, prior to the addition of the precipitating reagents. BSF was added up to 20 µM while HSA was added up to 100 µM. All readings were done in triplicates. The background readings associated with BSF or HSA in water alone were subtracted from each data point prior to their final analysis; BSF gave much higher background readings than HSA. After spectrophotometric reading, the suspensions were stored either at room temperature or 4°C for various periods of time before further centrifugation, washing, and resuspension, as outlined earlier, and processed accordingly for ultra-microscopy or SDS-PAGE.

### Lipid composition of NLP

To verify whether lipids would bind to NLP, NLP were prepared by mixing sequentially solutions of 0.25 M CaCl_2_, 0.25 M Na_2_CO_3_, and 0.25 M NaH_2_PO_4_, each at pH 7.4, with DMEM containing either 5% FBS or 5% HS. One ml FBS or HS was used. The precipitating ion groups were added each at a final concentration of 5 mM to a final volume of 20 ml. After incubation for 30 min, NLP were pelleted by centrifugation at 15,000×*g* for 10 min, washed twice in DMEM, and resuspended in 100 µl of HEPES buffer, of which 90 µl were used for lipid determination by the enzymatic-colorimetric analysis whereas the other 10 µl were washed twice with 1 ml distilled water, pelleted, and resuspended in 40 µl of 25 mM EDTA in water and used for agarose gel electrophoresis. The enzymatic-colorimetric methods used the 7600 Clinical Analyzer (Hitachi Science Systems, Tokyo, Japan). Triglycerides were measured using a lipase glycerol kinase enzymatic detection kit (Hoffman-La Roche, Nutley, NJ, USA). Total cholesterol was measured using a cholesterol esterase/peroxidase enzymatic detection kit (Wako Diagnostics, Richmond, VA, USA). HDL and LDL were measured using the cholesterol esterase/peroxidase enzymatic detection kits Cholestest®N HDL and Cholestest®N LDL (Sekisui Medical, Tokyo, Japan), respectively. Lipoprotein profiles of FBS NLP and HS NLP were analyzed by agarose gel electrophoresis using the SPIFE Lipoprotein detection kit (Helena, Beaumont, TX, USA). For agarose gel electrophoresis, 40 µl of FBS NLP or HS NLP resuspended in 25 mM EDTA were loaded on a 1% agarose gel and electrophoresed for 40 minutes. After migration, lipoproteins were stained with Fat Red 7B stain.
